# Transcriptional Profiles of Genes Related to Stress and Immune Response in Rainbow Trout (*Oncorhynchus mykiss*) Symptomatically or Asymptomatically Infected With *Vibrio anguillarum*

**DOI:** 10.3389/fimmu.2021.639489

**Published:** 2021-04-21

**Authors:** Zhi-Shuai Hou, Yuan-Ru Xin, Xiao-Dong Yang, Chu Zeng, Hong-Kui Zhao, Meng-Qun Liu, Mei-Zhao Zhang, Jeffrey G. Daniel, Ji-Fang Li, Hai-Shen Wen

**Affiliations:** ^1^ Key Laboratory of Mariculture (Ocean University of China), Ministry of Education (KLMME), Ocean University of China, Qingdao, China; ^2^ Department of Anatomy, Physiology, and Pharmacology, Auburn University College of Veterinary Medicine, Auburn, AL, United States

**Keywords:** rainbow trout, vibriosis, stress responses, immune functions, RNA-Seq

## Abstract

Rainbow trout (*Oncorhynchus mykiss*) is one of the most common aquaculture fish species worldwide. Vibriosis disease outbreaks cause significant setbacks to aquaculture. The stress and immune responses are bidirectionally modulated in response to the health challenges. Therefore, an investigation into the regulatory mechanisms of the stress and immune responses in trout is invaluable for identifying potential vibriosis treatments. We investigated the transcriptional profiles of genes associated with stress and trout immune functions after *Vibrio anguillarum* infection. We compared the control trout (CT, 0.9% saline injection), asymptomatic trout (AT, surviving trout with minor or no symptoms after bacteria injection), and symptomatic trout (ST, moribund trout with severe symptoms after bacteria injection). Our results showed activated immunomodulatory genes in the cytokine network and downregulated glucocorticoid and mineralocorticoid receptors in both AT and ST, indicating activation of the proinflammatory cytokine cascade as a common response in AT and ST. Moreover, the AT specifically activated the complement- and TNF-associated immune defenses in response to *V. anguillarum* infection. However, the complement and coagulation cascades, as well as steroid hormone homeostasis in ST, were disturbed by *V. anguillarum*. Our studies provide new insights toward understanding regulatory mechanisms in stress and immune functions in response to diseases.

## Highlights

- Asymptomatic and symptomatic trout mounted different immune responses
- *V. anguillarum* infection activated the proinflammatory cytokine cascade
- The complement- and TNF-related immune defenses were specifically activated in asymptomatic trout
- Diverse functions were identified among three novel *c3-1 subtypes*

**Graphical Abstract:**
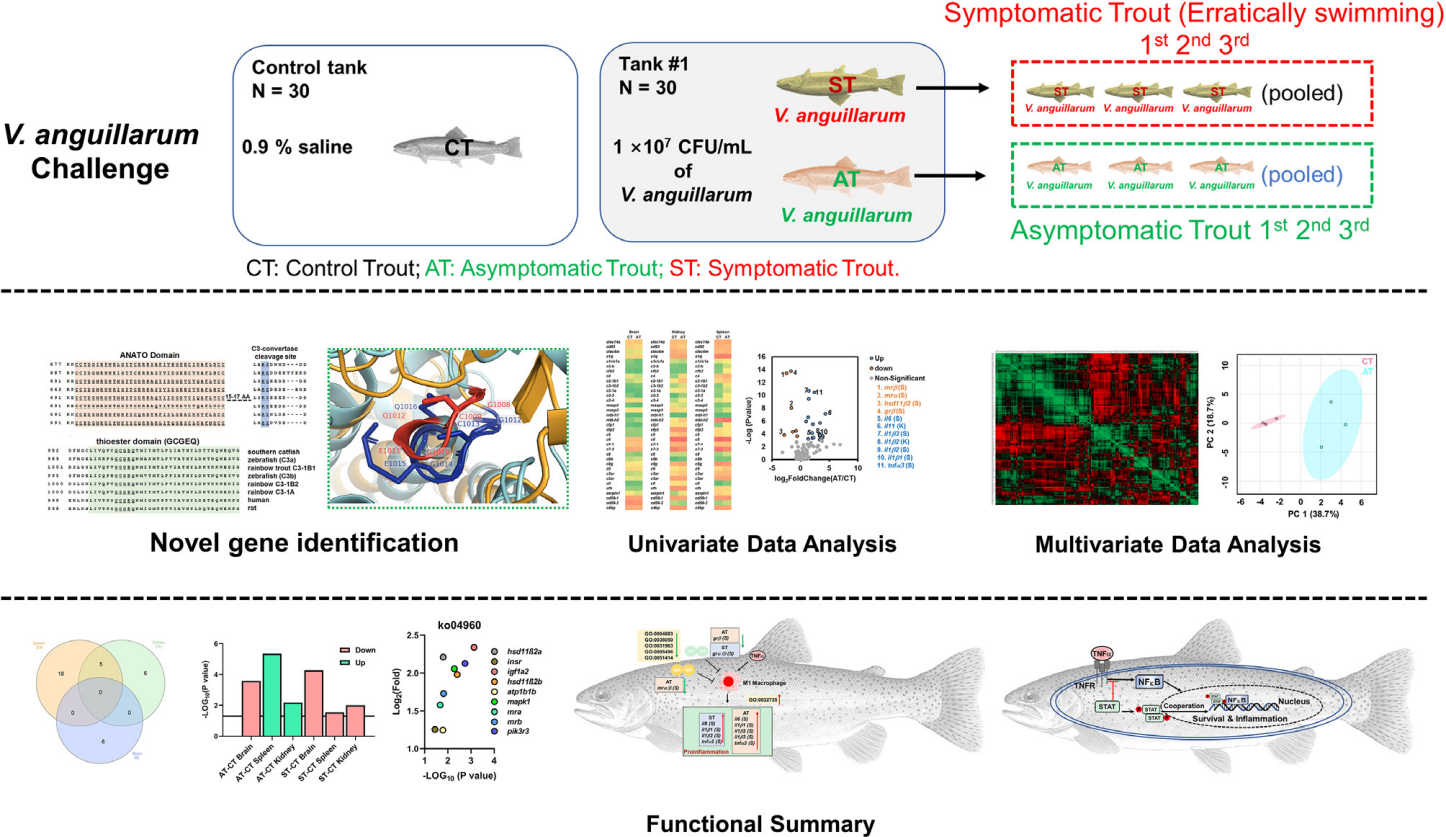


## Introduction

Teleosts have to cope with various challenges, including the diversity of the potential environmental stimuli and pathogen load (1, 2). Although teleosts respond differently to stressors and the immune responses also remain species-specific, environmental and aquaculture insults can trigger defensive reactions of fish, including the activation of the stress response (3, 4). Based on energy balance, the stress response results in energy redistribution with the ultimate purpose to restore homeostasis, thus saving the energy that is not necessary to survive and enabling fishes to prepare for “fight” or “flight” (5–7). For example, a slightly activated stress response could enhance immune competence (fight), while a prolonged stress response suppresses immune function (flight) (8).

Cortisol and its receptors [glucocorticoid receptor (GR) and mineralocorticoid receptor (MR) (9)] play an important role in regulating crosstalk between the stress response and immune networks. Activation of the GR (or MR) may serve as an early danger alarm and enable the immune system to prepare for the fight against health challenges (10, 11). Moreover, GR (or MR) activation modulates the leukocyte-regulated immune responses and negotiates the initiation and efficacy of immune functions (1). Inflammation serves as the first step of immunomodulation in response to infection or irritation (12). Proinflammatory cytokines, such as interleukin 1 (IL-1) and tumor necrosis factor α (TNFα) (5), act as an important defense mechanism against pathogens. The stress response typically regulates the immune response by suppressing the synthesis and release of proinflammatory cytokines in both mammals and teleosts (13–15).

In the mid-1980s, a series of papers published in *Science* showed that proinflammatory cytokines act as stress-response regulators [reviewed in (16)]. Another previous study showed that cytokines regulate stress responses in mammals by decreasing GR expression, blocking GR translocation, and disrupting GR-DNA binding in the nucleus (17). In response to pathogen infection, the homeostatic interaction between the stress response and cytokine-induced inflammation in teleosts is more complicated, showing no negative or positive correlation among various teleosts. For example, the stress response (mimicked by cortisol) does not affect cytokine gene expression in rainbow trout (*Oncorhynchus mykiss*); however, the stress response did reduce the stimulated gene expression of all cytokines in gilthead sea bream (*Sparus aurata*) (11). In the European sea bass (*Dicentrarchus labrax*), genes associated with glucocorticoid synthesis and inflammatory responses are simultaneously upregulated after *Vibrio anguillarum* infection (5). These studies indicate that the interplay between stress and immune responses is differentially regulated in various teleost species.

In addition to the cytokines, the complement cascade is also involved in immunomodulation in response to pathogen invasion. The complement system, which was identified a century ago, is the most ancient and essential immune system component [reviewed in (18–20)]. The complement system is the first immune response against invading pathogens and orchestrates the subsequent immunological and inflammatory processes associated with detection, destruction, and elimination of the microbial intruders [Reviewed in (18–20)]. The mammalian complement repertoire includes ~35 plasma (hydrophilic)- and membrane (hydrophobic)-bound complement proteins (21). Although the mammalian complement system can be activated by the classical, lectin, or alternative pathways, all three pathways share the common step of activating the component C3 (18). The physiological functions and signaling cascades of the complement system are mostly conserved between mammals and teleosts (22, 23). An activated complement system will release complement protein fragments that typically kill the microbial intruders and orchestrate immunological and inflammatory homeostasis (22). Early studies in rainbow trout showed that the complement system accounts for resistance to furunculosis or vibriosis (24, 25). These two highly contagious diseases cause excessive trout mortality, which leads to significant aquacultural economic loss.

Infectious diseases are constant threats to aquaculture and larviculture, causing significant financial losses due to high infectivity and mortality (11). *V. anguillarum*, the causative agent of vibriosis, is a gram-negative bacteria that causes severe, frequently deadly hemorrhagic septicemia in teleosts (26, 27). The previous studies showed that fish exhibit higher individual variations in response to pathogen infection (28–30). Genetic factors that favor the survival of asymptomatic individuals could be used as targets for selecting disease-resistant fish, thus reducing economic loss from infectious disease (31). Although accumulating studies have been focused on generating disease (or stress)-resistant fish strains, the mechanisms remain largely unknown (31, 32). Investigation of the target genes and pathways associated with disease-resistant could potentially provide molecular markers for genetic breeding.

**Figure 1:**
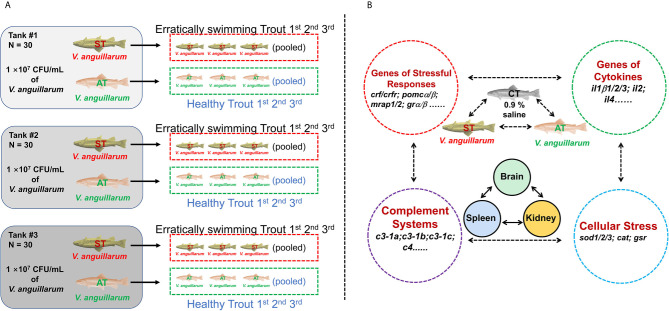
Experimental setup. **(A)** 90 trout were randomly and equally distributed into three tanks and then challenged with *V. anguillarum* with 10^7^ CFU/ml. The first three erratically swimming individuals with severe symptoms in tank #1 were pooled as sample #1 of the symptomatic trout (ST). After 120 h post-challenge, the three surviving individuals with minor or no symptoms were pooled as sample #1 of the asymptomatic trout (AT). Likewise, sample #2 of ST and AT, as well as sample #3 of ST and AT, were collected from tank #2 and tank #3, respectively. The control trout were injected with 0.9% NaCl and then sampled with the same protocol. **(A)** was partly adapted from **Figure 1** in our previous paper (33)]. **(B)** Based on previous studies (35–40), genes in the brain, kidney, and spleen associated with stress and immune functions were investigated in CT, ST, and AT.

Rainbow trout (*Oncorhynchus mykiss*) is one of the most common aquaculture fish species worldwide (Food and Agriculture Organization of the United Nations); however, the trout industry is severely affected by vibriosis (27). In this study, the RNA-Seq datasets were retrieved from our previous studies (33, 34), and we analyzed a total of 27 RNA-Seq libraries. Briefly, we investigated control trout (0.9% saline-injection), asymptomatic trout (AT; surviving trout with minor or no symptoms after *V. anguillarum* injection), and symptomatic trout (ST; moribund trout with severe symptoms after *V. anguillarum* injection). The brain, kidney, and spleen were collected for RNA-Seq. Previous studies in trout revealed important genes involved in regulating stress responses and immune functions (35–40); therefore, we targeted these candidate genes (**Figure 1**). Our studies showed that complement- and TNF-associated immune defenses were specifically activated in AT. Our studies provide new insights into the stress-immune network in response to pathogen infection in trout and provide potential molecular markers for genetic breeding of disease-resistant trout populations.

## Materials and Methods

### Ethics Statement

Experiments in this study were conducted in accordance with Guidelines of Animal Research and Ethics Committees of Ocean University of China (Permit Number: 20141201), U.K. Animals (Scientific Procedures) Act, 1986 and associated guidelines, EU Directive 2010/63/EU for animal experiments, and use of laboratory animals (NIH Publications No. 8023, revised 1978) National Institutes of Health Guide for the Care and Use of Laboratory Animals (NIH publication no. 8023, revised 1978). No endangered or protected animal species were used. The effects of sex were not considered because trout juveniles are sexually immature.

### Animals

Rainbow trout juveniles were obtained from Linqu Salmon and Trout Aquatic Breeding LLC (Weifang, Shandong, China). These juveniles were from the same full-sibling family batch and spawned on the same day with synchronized development. Trout were acclimatized for 14 days in indoor cuboidal tanks equipped with a water pump, chiller system, sand filter, and biofilter at the Experimental Fish Facility in Key Laboratory of Mariculture, Ocean University of China. According to the Standards of Linxia Salmon and National Trout Elite Breeding and Protection Farm (Linxia, Gansu, China, Approved by Department of Agriculture, China, 2009), trout were cultured at ~16°C and ~7 mg/L of dissolved oxygen. Trout were fed a commercial pellet twice a day at 7% of total body weight.

### V. anguillarum

The *V. anguillarum* strain was obtained from the Laboratory of Pathology and Immunology of Aquatic Animals, Ocean University of China (41, 42). The bacteria were grown overnight at 28°C in 2216E medium. The bacterial suspension was then centrifuged and resuspended with 0.01 M phosphate-buffered saline (PBS, pH = 7.2). *V. anguillarum* suspension density was adjusted to serial dilutions for preliminary testing: 10^9^, 10^8^, or 10^7^ colony forming units (CFU)/ml (33).

### Experimental Design

This manuscript used the same RNA-Seq samples previously described in two papers evaluating the growth hormone and insulin-like growth factor axes, as well as the caspase gene family in rainbow trout (33, 34). Previous studies showed 10^7^ to 10^9^ CFU/ml of *V. anguillarum* could cause vibriosis in rainbow trout and other teleosts (5, 41–43). Our published paper further showed that *V. anguillarum* of 10^7^ CFU/ml at 20°C exhibited mild to moderate symptoms of vibriosis disease with a relatively lower mortality (33). Therefore, trout were challenged by 10^7^ CFU/ml of *V. anguillarum* at 20°C. In the control group, 90 trout were randomly distributed into three tanks, with 30 trout in each tank. The control trout (CT) were intraperitoneally injected with 200 μl physiological saline (saline-challenged, 0.9% NaCl). In the challenged group, 90 trout were equally and randomly distributed into three tanks. Trout of the challenged group were challenged by intraperitoneal injection of 200 μl *V. anguillarum* (10^7^ CFU/ml). In challenged groups, the first three erratically swimming moribund trout showing severe symptoms, such as hemorrhage in fins, in tank #1 were pooled as sample #1 of the symptomatic trout (ST). After 120 h post-challenge, the three surviving trout with minor or no symptoms were pooled as sample #1 of the asymptomatic trout (AT) (**Figure 1**). Likewise, sample #2 of ST and AT, as well as sample #3 of ST and AT, were collected from tank #2 and tank #3, respectively (**Figure 1**). Trout were anesthetized by MS-222 (35–45 mg/L [ppm]) before sampling. Biological samples of organs and tissues (brain, spleen, kidney, liver, and gill) were isolated and stored at −80°C for further analysis.

### RNA-Seq Analysis

A total of 27 libraries [3 tissues (brain, kidney, spleen) × 3 replicated samples (each sample contained three pooled individuals × 3 treatment groups] was constructed *via* the TruSeq™ RNA Sample Prep Kit (Illumina, CA, USA). This study used the same RNA-Seq data with our previously published paper (33, 34), but we focused on different functional genes and used various analyses. The sequence reads are available from the NCBI sequence read archive (SRA) with the accession number of PRJNA667799.

### Novel Gene(s) Identification

The amino acid sequences of trout novel C3-1 proteins, and zebrafish (*Danio rerio*), southern catfish (*Silurus meridionalis*), rat (*Rattus norvegicus*), and human (*Homo sapiens*) C3 proteins were used for the phylogenetic analysis and sequence alignment. Phylogenetic analyses were plotted using the Neighbor-joining (N-J) method *via* MEGA 7, with 1000 bootstrap replications for phylogeny. The SWISS-MODEL between trout and mammalian C3 proteins was generated using the SWISS-MODEL (https://swissmodel.expasy.oAT/) (44, 45). The mammalian C3 with an intact thioester at 3Å resolution [PDB ID: 2B39 (46)] was used as the template. Comparison of the domains between trout and mammalian C3 and the cartoon, stick, and sphere structures of the proteins were generated with the PyMOL software package (47, 48).

### Statistical Analysis

Based on published papers on biomedical and fishery studies (49–51), the peak intensity tables of selected genes were uploaded to the websites of MetaboAnalyst and NetworkAnalyst (ATtps://www.xialab.ca/tools.xATml) for data processing and analyses (52). The uploaded data (count normalized by DESeq2 package in the R software (53)) were performed by sum normalization, thus obtaining the belt data (Poisson) distribution for further statistical analysis (**Figure S1**). In the multivariate analysis module of MetaboAnalyst, the normalized data were then subject to principal component analysis (PCA) and partial least squares discriminant analysis (PLSDA) for pattern discovery (**Figure S1**). Genes of each pairwise comparison (ST/CT, AT/CT, or AT/ST) were selected to create a heatmap (Based on log_10_(normalized count+1)) and correlation analysis (with Pearson’s correlation) (51).

## Results

### Differentially Expressed Genes Between ST and CT

**Figure 2:**
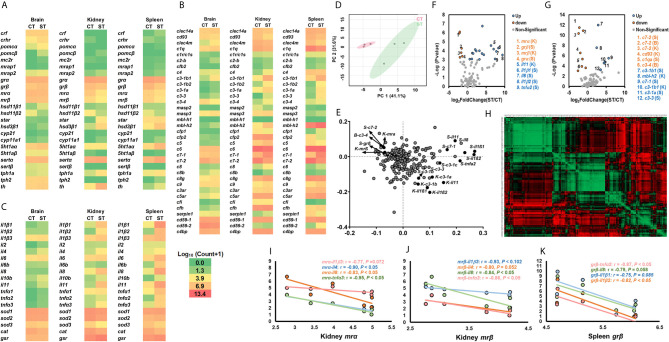
Transcriptional profiles of genes in stress and immune functions between ST and CT. **(A–C)** The heatmap of genes related to the stress response **(A)**, cytokines and cellular functions **(B)**, and the complement system **(C)**. The heatmap is generated by the values of log_10_ (normalized count+1). The red shows high expression, and green shows low expression. More details are shown in **Table 1**. Basal gene expression is shown in **Figure S5**. **(D, E)** PCA **(D)** and loading plots **(E)** of genes related to the stress response, cytokines, cellular functions, and the complement system. The red dots show the vector of overall gene expression in CT, and the green dots show the vector of overall gene expression in ST. Details of the loading plot are shown in **Table S1**. **(F, G)** Volcano plots of genes of the stress response and cytokine **(F)**, and the complement system **(G)**. Negative and positive Log_2_FoldChange show down-regulation and upregulation, respectively (ST vs. CT). More details are shown in **Table 1** **. (H–K)** Correlations of genes related to the stress response, cytokines, cellular functions, and the complement system. The detailed view of **(H)** is shown in **Figure S2**. Gene abbreviations are shown in **Table 1**.

The heatmap displayed the transcriptional profile of genes associated with the stress response, cytokines and cellular functions, and the complement system between ST and CT (**Figures 2A–C**). The overall transcriptional profiles of target genes in ST and CT in response to *V. anguillarum* infection were summarized by PCA (**Figure 2D**). Red dots show the vector containing overall gene expression in CT, and green dots showed the vector containing overall gene expression in ST. Separated PCA vectors were present, indicating that the *V. anguillarum* infection resulted in different profiles of genes associated with the stress response, cytokines and cellular functions, and the complement system between ST and CT (**Figure 2D**). The loading plot of PCA shows the genes exerting stronger influences on PCA analysis (**Figure 2E**, points far away from the zero point, **Table S1**).

The volcano plots showed that, compared to CT, the ST showed significantly downregulated kidney *mrα, mrβ*, *c7-2*, and *cd93*, and spleen *grα, grβ, c7-2*, and c1qa, and brain *c7-2* and *c3-4* (**Figures 2F, G**). Compared to CT, the kidney *il11, mbl-h2*, and *c3-1b1*, and spleen *il1β1, il1β2, il8, tnfα2, c3-1a, c3-1b1*, and *c3-3* were significantly upregulated in ST (**Figures 2F, G**). The genes showed in volcano plots were labeled in the loading plot (**Figure 2E**).

The correlation analysis of all target genes is depicted using a heatmap (**Figure 2H** and **Figure S2**). The Pearson correlation coefficients showed that the kidney *mrα* or *mrβ* exhibited strong negative relationships with the cytokines of *il1β3, il4, il8*, and *tnfα3* (**Figures 2H–J**). The spleen *grβ* showed negative relationships with *il1β1, il1β2, il8*, and *tnfα2* (**Figures 2H, K**).

### Differentially Expressed Genes Between AT and CT

**Figure 3:**
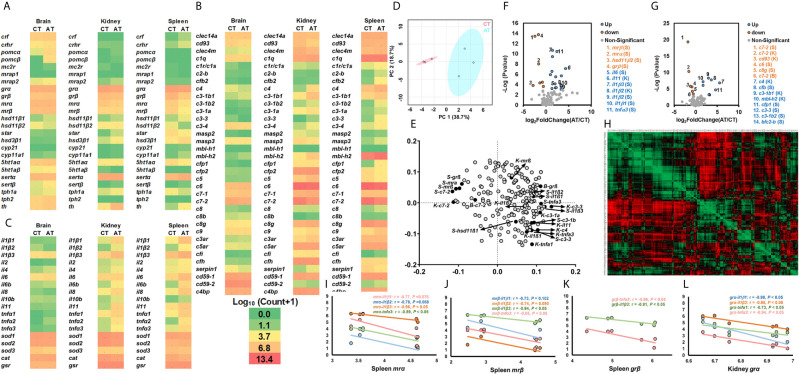
Transcriptional profiles of genes in stress and immune functions between AT and CT. **(A–C)** The heatmap of genes of the stress response **(A)**, cytokines and cellular functions **(B)**, and the complement system **(C)**. The heatmap is generated by the values of log_10_ (normalized count+1). The red shows high expression, and green shows low expression. More details are shown in **Table 1**. Basal gene expression is shown in **Figure S5**. **(D, E)** PCA **(D)** and loading plots **(E)** of genes related to the stress response, cytokines, cellular functions, and the complement system. The red dots show the vector of overall gene expression in CT, and the blue dots show the vector of overall gene expression in AT. Details of the loading plot are shown in **Table S2**. **(F, G)** Volcano plots of genes of the stress response and cytokines **(F)**, and the complement system **(G)**. Negative and positive Log_2_FoldChange show down-regulation and upregulation, respectively (AT vs. CT). More details are shown in **Table 1**. **(H–L)** Correlations of genes related to the stress response, cytokines, cellular functions, and the complement system. The detailed view of **(H)** is shown in **Figure S3**. Gene abbreviations are shown in **Table 1**.

The transcriptional profiles of genes involved in the stress response, cytokines, cellular functions, and the complement system between AT and CT were shown by heatmap (**Figures 3A–C**). Separated PCA plots indicate that genes related to cytokines, the stress response, cellular functions, and the complement system were differently expressed in AT and CT (**Figure 3D**). The loading plot showed the genes significantly involved in the separated PCA plots (**Figure 3E**, points far away from the zero point, **Table S2**).

The volcano plots showed that, compared to CT, the AT showed downregulated kidney *c7-2, cd93*, and spleen *mrα, mrβ, grβ, hsd11β2, c7-2, c6*, and *c8g*, and brain *c7-2* (**Figures 3F, G**). The AT exhibited significantly upregulated kidney *il1β2, il11, c4*, and *mbl-h2*, and spleen *il1β1, il1β2, il1β3, il6, tnfα3, cfb, cfp1, c3-1b2, c3-3*, and *bcf2-b* (**Figures 3F, G**). These genes were highlighted in the loading plot (**Figure 3E**).

Heatmap showing the Pearson correlation coefficients of genes (**Figure 3H** and **Figure S3**). Pearson correlation coefficients showed that the spleen *mrα* or *mrβ* exhibited strong negative relationships with the cytokines of *il1β1, il1β2, il1β3* and *tnfα3* (**Figures 3H–J**). The spleen *grβ* exhibited negative Pearson correlation coefficients with *il1β3*, and *tnfα3* (**Figures 3H, L**), while the kidney *grα* showed negative relationships with *il1β1, il1β3, tnfα1* and *tnfα3* (**Figures 3H, K**).

### Differentially Expressed Genes Between ST and AT

**Figure 4:**
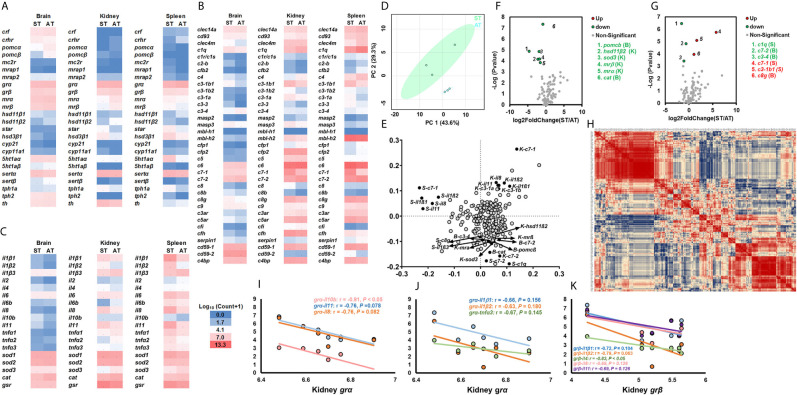
Transcriptional profiles of genes in stress and immune functions between ST and AT. **(A–C)** The heatmap of genes of the stress response **(A)**, cytokines and cellular functions **(B)**, and the complement system **(C)**. The heatmap is generated by the values of log_10_ (normalized count+1). The red shows high expression, and the blue shows low expression. More details are shown in **Table 1**. Basal gene expression is shown in **Figure S5**. **(D, E)** PCA **(D)** and loading plots **(E)** of genes related to the stress response, cytokines, cellular functions, and the complement system. The blue dots show the vector of overall gene expression in AT, and the green dots show the vector of overall gene expression in ST. Details of the loading plot are shown in **Table S3**. **(F, G)** Volcano plots of genes related to the stress response and cytokines **(F)**, and the complement system **(G)**. Negative and positive Log_2_FoldChange show down-regulation and upregulation, respectively (ST vs. AT). More details are shown in **Table 1** **. (H–K)** Correlations of genes related to the stress response, cytokines, cellular functions, and the complement system. The detailed view of **(H)** is shown in **Figure S4**. Gene abbreviations are shown in **Table 1**.

We compared the overall gene expression between ST and AT by heatmap (**Figures 4A–C**). In PCA plots, vectors showing gene expression in ST were separated from those showing gene expression in AT, demonstrating differential gene expression between ST and AT in response to *V. anguillarum* infection (**Figure 4D**). The loading plot showed key genes resulting in discrimination and stronger influences on PCA vectors (**Figure 4E**, points far away from the zero point, **Table S3**).

Volcano plots showed the expression of genes (kidney *hsd11β2, sod3, mrα*, and *mrβ*, and spleen *c1qa*, and brain *pomcβ, cat, c3-4*, and *c7-2*) in ST were significantly lower than those of AT (**Figures 4F, G**). Compared to AT, ST showed upregulated gene expression of spleen *c7-1* and *c3-1b1* and brain *c8g* (**Figures 4F, G**). The Pearson correlation coefficients of target genes are indicated by a heatmap (**Figure 4H** and **Figure S4**). The kidney *grα* and *grβ* showed strong negative relationships with the cytokines of *il10b* and *il4*, respectively (**Figures 4H, I, K**).

### Identification of Novel *c3* Gene Subtypes

**Figure 5:**
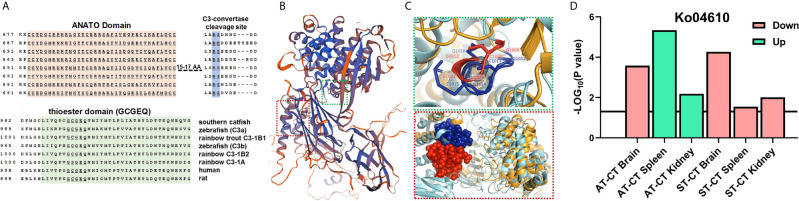
Identification of *c3* gene subtypes and characterization of the enriched pathway. **(A)** Alignment of novel trout *c3* gene subtypes to teleost and mammalian species (ANATO and thioester domains and C3-convertase cleavage site are shown, and the whole sequence alignment is shown in **Figure S7**. The *c3-1a*: LOC110489008, XP_021417220.2; *c3-1b1*: LOC110489027, XP_021417252.2; *c3-1b2*: LOC110517348, XP_021451128.2. **(B)** The SWISS-MODEL of trout C3 to mammalian C3 (PDB ID: 2B39). Blue shows the conserved motif, and red shows the less conserved motif between trout and mammalian C3. Red and green boxes, respectively, mark the ANATO and thioester domains. Parameters of SWISS-MODEL template (local quality estimate and Model-Template alignment) of trout C3 and mammalian C3 are shown in **Figure S8**. **(C)** Comparison of the ANATO (red) and thioester (green) domains between trout and mammalian C3. Cyan cartoon shows the mammalian C3, and khaki cartoon shows the trout C3. Red labels, sticks, and spheres show the mammalian C3, and blue labels, sticks, and spheres show the trout C3. The 3D cartoon for the comparison of mammalian and trout C3 is shown in **Figure S9** (whole structure). **(D)** The enriched KEGG pathway involved in complement systems in brain, kidney and spleen of CT, ST, and AT (Ko04610, complement and coagulation cascades).

We identified three novel *c3* gene subtypes in RNA-Seq data. Based on the alignment of the amino acid sequences, these three C3 proteins showed the conserved functional domains, including the ANATO domain, thioester domain, and C3-convertase cleavage site (**Figure 5A** and the whole sequences alignment are shown in **Figure S7**). Based on mammalian C3 (PDB ID: 2B39), the SWISS-MODEL illustrated conserved motifs between trout and mammalian C3 with blue cartoons (**Figure 5B** and **Figures S7, S8, S9A, S9B, S9C**). Red and green boxes mark the ANATO and thioester domains, respectively (**Figures 5B, C**). The comparison of the thioester (green) and ANATO (red) domains between trout and mammalian C3 are shown in cartoons (**Figure 5C** and **Figures S9B, S9C**). The conserved amino acid sequences of GCGEQ in thioester domain were labeled (**Figure 5C**, top figure). The locations were adjacent, and the identities were identical for both GCGEQ sequences of mammalian and trout C3 (**Figure 5C**, top figure; **Figure S9C**). Likewise, the ANATO domains of both mammalian and trout C3 are similarly organized, and their amino acid sequences were highly identical (**Figure 5C**, bottom figure; **Figure S9B**). The gene expression levels of three *c3* were shown (**Figures S9D–F**). Compared to CT, the ST showed significantly upregulated *c3-1a* and *c3-1b1* expression in the kidney and spleen (**Figures S9D, E**).

### Functional Enrichment Analysis of DEGs

Compared to CT, the AT showed upregulated Ko04610 (complement and coagulation cascades) in the kidney and spleen (**Figure 5D**). In contrast, the ST showed a downregulated Ko04610 pathway in the brain, kidney, and spleen (**Figure 5D**). No significant changes in the Ko04610 pathway were observed in the kidney and spleen between AT and ST. The overlapping genes in the Ko04610 pathway are shown in Venn diagrams (**Figures S9G, S9H**), and their expression levels among CT, AT, or ST were shown by heatmap (**Figures S9I–S9L**).

**Figure 6:**
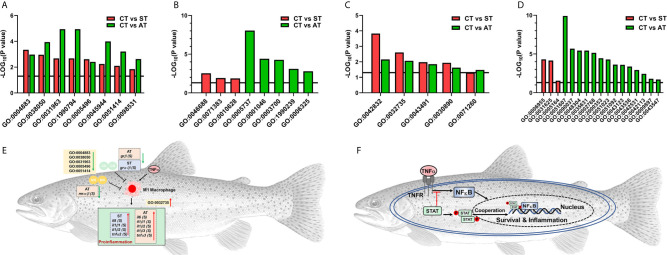
Enriched GO terms associated with gr subtypes **(A, B)** or associated with tnfa subtypes **(C, D)**. **(A)** The enriched GO terms shared in comparisons of CT vs. ST and CT vs. AT. **(B)** The enriched GO terms specifically identified in CT vs. ST or CT vs. AT. **(C)** The enriched GO terms shared in comparisons of CT vs. ST and CT vs. AT. **(D)** The enriched GO terms that are specifically identified in CT vs. ST or CT vs. AT. Details for GO terms annotation are shown in **Table 2**. **(E)** M1 macrophage polarization potentially activates proinflammatory cytokine cascade response. **(F)** The phosphorylated STAT dimer enhances TNFα-regulated immunomodulation, thus enabling the trout in AT to fight off the pathogen infection.

We showed *grα* and *grβ* were shared in the list of DEGs between groups of CT and ST or CT and AT. In the gene ontologies (GO) terms involved in *grα* and *grβ*, 8 GO terms were shared between the comparisons of CT and ST or CT and AT (**Figure 6A**, details in **Table 2**). Three GO terms were specifically enriched in the comparison of CT and ST (**Figure 6B**, details in **Table 2**), and five GO terms were specifically enriched in the comparison of CT and AT (**Figure 6B**, details in **Table 2**). Likewise, *tnfα* subtypes were shared in the DEGs list between CT and ST or CT and AT. Five GO terms associated with *tnfα* were both identified between the comparisons of CT and ST or CT and AT (**Figure 6C**, details in **Table 2**). Three GO terms were specifically enriched in the comparison of CT and ST (**Figure 6D**, Details in **Table 2**), and 14 GO terms were specifically enriched in the comparison of CT and AT (**Figure 6D**, details in **Table 2**).

**Figure 7:**
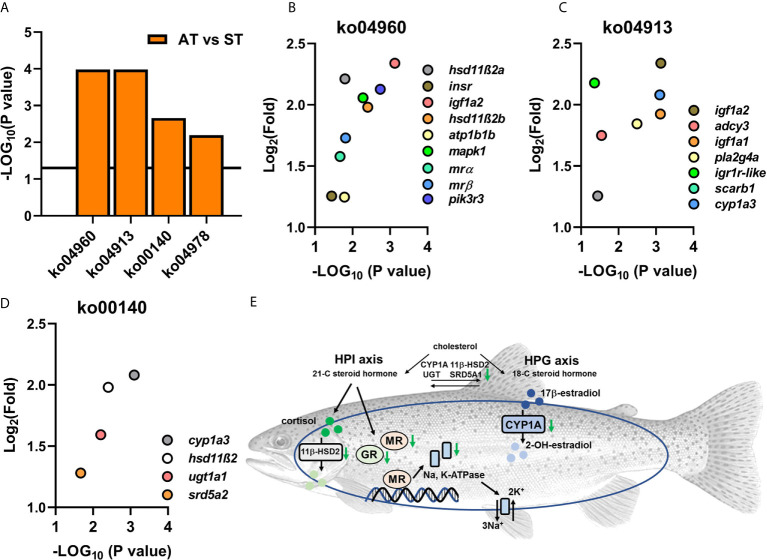
Enriched KEGG pathways **(A)** and transcriptional levels of DEGs from enriched pathways **(B–D)**. **(A)** The enriched KEGG pathways in comparisons of AT vs. ST. **(B)** Transcriptional levels of DEGs from enriched KEGG pathway of ko04960 (aldosterone-regulated hydromineral balance). **(C)** Transcriptional levels of DEGs from enriched KEGG pathway of ko04913 (steroidogenesis). **(D)** Transcriptional levels of DEGs from enriched KEGG pathway of ko00140 (steroid hormone biosynthesis). **(E)** The enriched KEGG pathways showed endocrine dyshomeostasis resulting from *V. anguillarum* infection might serve as a lethal factor in trout of ST.

Genes of *mrα* and *mrβ* were identified in the list of DEGs between the comparison of ST and AT. Based on the KEGG database, four pathways that are associated with functions of steroid hormones were enriched (**Figure 7A**), including ko04960 (aldosterone-regulated sodium reabsorption, **Figure 7B**), ko04978 (mineral absorption), ko00140 (steroid hormone biosynthesis, **Figure 7C**) and ko04913 (ovarian steroidogenesis, **Figure 7D**).

## Discussion

**Figure 8:**
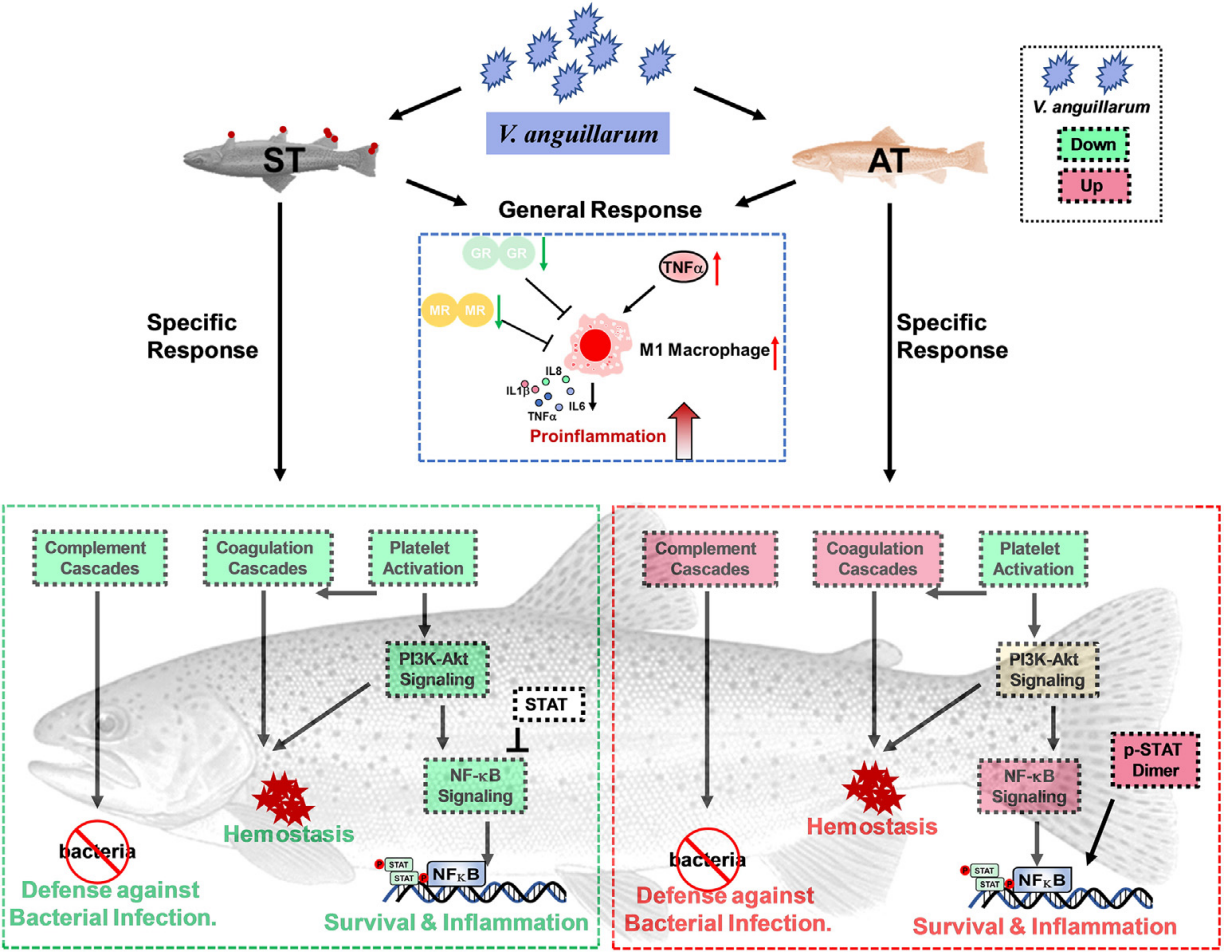
Putative pathways involved in defense mechanism, hemostasis, and inflammatory responses based on RNA-Seq signatures.

Several studies have already focused on reactions of stress- and immune-related functions to *V. anguillarum* infection in teleosts, showing the teleosts exhibit species-specific modulations (1, 5, 11). Therefore, we evaluated stress response and immune network changes in trout after *V. anguillarum* infection. Previous studies evaluated the immunomodulation of European sea bass and flounder (*Paralichthys olivaceus*) with *V. anguillarum* concentration of 10^7^ CFU/ml (5, 54). Consistently, our preliminary trial showed trout challenged by 10^7^ CFU/ml of *V. anguillarum* exerted mild to moderate symptoms compared to trout infected by 10^8^ or 10^9^ CFU/ml of *V. anguillarum* (33). In brief, trout challenged by 10^7^ CFU/ml of *V. anguillarum* began to die within 24 h after challenge and the mortality is around 20% within 120 h after challenge (33). Moreover, the RNA-seq and qPCR data consistently showed the ST and AT exerted different expressions of genes in caspase family (34). For example, ST showed higher up-regulated *casp8*, which is involved in apoptosis regulation, pathogen detection and immunomodulation (34). In this study, based on multivariate analysis of PCA, significant differences in the transcriptional profiles of stress and immune-related genes were observed in trout between the pairwise comparisons of CT, AT, and ST (**Figures 2D**–**4D** and **Figure S1**). The analysis of gene expression and pathway enrichment showed that the proinflammatory cytokine cascade response, which is potentially caused by M1 macrophage polarization, is activated in both AT and ST (**Figures 6** and **8**). However, the complement system showed phenotype-specific responses between AT and ST (**Figures 6** and **8**).

### Complement System

The C3 serves as a major acute-phase protein (55). The expression of *c3* gene subtypes is significantly upregulated in response to bacterial or LPS stimulation in multiple teleosts, including the dojo loach (*Misturnus anguillicaudatus*), rainbow trout, southern catfish (*Silurus meridionalis*), and grass carp (*Ctenopharyngodon idella*) (56–59). Consistently, our study found that the trout *c3* gene subtypes showed upregulation in responses to *V. anguillarum* infection. Salmonidae species, such as trout and salmon, experienced four rounds of genome duplication. Consequently, the genetic expansions are characterized by duplicated functional gene copies (paralogs) in Salmonidae fishes (60, 61). Previous studies identified multiple trout *c3* subtypes (*c3-1*, *c3-3*, and *c3-4*) with functional diversity (62, 63). Our study identified three novel subtypes within *c3-1* (*c3-1a*, *c3-1b1*, and *c3-1b2*) (**Figure 5** and **Figures S7–S9**). These genes exhibited conserved sequence identity but specific expression patterns in responses to *V. anguillarum* infection (**Figure 5** and **Figures S7–S9**), indicating that these genes can encode bioactive proteins with diversity in functions.

The complement system served as a major governor of inflammatory responses (64). The homeostasis of inflammatory reactions plays a vital role in modulating health balance. Either inefficient or overactive activation of the complement system could disturb the homeostasis, which is detrimental for health balance (64, 65). Compared to CT, the kidney and spleen of ST exhibited downregulated complement cascades (Ko04610). Previous studies in mice indicated that the inefficient activation of complement cascades might be associated with increased susceptibility to infectious diseases (64, 66). Therefore, the ST showed severe symptoms in response to *V. anguillarum* infection. The complement and coagulation cascades belong to a complex inflammation regulatory network (67, 68). In most of the pathophysiological processes, both the complement and coagulation cascades are activated simultaneously (69). Consistent with the downregulated complement cascades, key genes in coagulation cascades were downregulated in the kidney and spleen of ST, including *vwf* subtypes (von Willebrand factor), *α2m* (*α*-2-macroglobulin), and *f13a* (Coagulation factor XIII A chain). The ST also showed downregulated platelet activation (ko04611, **Figure S10**). The downregulated coagulation cascades and platelet activation probably caused severe hemorrhages in the fins, kidneys, and other visceral masses in ST, all of which were lethal to the trout. Studies in biomedical and fishery sciences showed healthy individuals could efficiently regulate the complement system, thus not only preventing the complement(s) exhaustion but also enabling the complement(s) to restore (21, 70). However, the moribund trout might fail to efficiently regulate the complement system. The complement exhaustion further reduced the defense to pathogen infection and eventually caused the worse outcomes (death) (71, 72).

The complement system can activate the innate immune system and thus play an essential role in linking the innate and adaptive systems in mammals and teleosts (18, 35, 55, 73). The AT showed upregulated complement and coagulation cascades, enabling the AT to fight the inflammatory pathogenesis and prevents life-threatening bleeding (69). Consistently, AT had higher *fga* and *fgb* expression (*fga and fgb*: fibrinogen *α*/*β* chain, which has a significant function in hemostasis, **Figure S10**). Based on these pieces of evidence, we propose that the different responses of complement and coagulation cascades might link to varying phenotypes of trout in response to *V. anguillarum* infection. A recent study showed that complement cascades serve as a bridge between immunomodulation in trout in response to bacterial infection (74), consistent with what we found in our study.

### Cytokine Networks

The cytokine networks govern the normal development and physiology in animals, and dysregulations of cytokine networks are involved in pathophysiological alternations (75). In humans, the IL1 serves as the most potent endogenous pyrogens in organisms affected by infectious diseases (76, 77). Likewise, IL1 plays an apical role in initiating inflammatory responses in teleosts (78), and *V. anguillarum* infection results in significantly upregulated *il1β* in teleosts, including Atlantic cod (*Gadus morhua*), sea bream, and European sea bass (79–82). Our study showed that ST and AT exhibited significantly upregulated *il1β* subtypes (**Figures 2** and **3**, **Table 1**). ST and AT also showed upregulated *tnfα* subtypes (**Figures 2** and **3**, **Table 1**), which was consistent with a previous study showing that the functions of IL1 and TNF largely overlap in teleosts (83). Indeed, the IL1 and TNF work synergistically, and the TNF usually acts as the first cytokine to follow an IL1 surge in an inflammatory response (83). Like IL1β, IL11 could regulate a series of important immunomodulatory effects by affecting proliferation and differentiation of hematopoietic progenitors, thus serving as a multifunctional modulator (84, 85). Studies showed kidney *il11* was significantly upregulated in response to bacterial pathogens in golden pompano (*Trachinotus ovatus*) (86), which is in line with our results (**Figures 2** and **3**, **Table 1**).

**Table 1:** Gene list of **Figures 2**–**4**.

Gene	Full Name	Function Description		Gene ID	Expression patterns between the pairwise comparisons		
					ST vs. CT	AT vs. CT	ST vs. AT
*pomcβ* (B)	pro-opiomelanocortin β	stimulate the adrenal glands to release cortisol.	The stress response	NM_001124719.1			down
*mrα* (K)	mineralocorticoid receptor α	mineralocorticoids/glucocorticoid receptor		NM_001124730.1	down		down
mr*β* (K)	mineralocorticoid receptor β	mineralocorticoids/glucocorticoid receptor		NM_001124740.1	down		down
*mrα* (S)	mineralocorticoid receptor α	mineralocorticoids/glucocorticoid receptor		NM_001124730.1		down	
*mrβ* (S)	mineralocorticoid receptor β	mineralocorticoids/glucocorticoid receptor		NM_001124740.1		down	
*grα* (S)	glucocorticoid receptor α	regulate inflammation, cellular proliferation, and differentiation		NM_001124730.1	down		
*grβ* (S)	glucocorticoid receptor β	regulate inflammation, cellular proliferation, and differentiation		NM_001124482.1	down	down	
*hsd11β2* (S)	corticosteroid 11β dehydrogenase isozyme 2	catalyzes the conversion of cortisol to the inactive metabolite cortisone		NM_001124218.1		down	
*hsd11β2* (K)	corticosteroid 11β dehydrogenase isozyme 2	catalyzes the conversion of cortisol to the inactive metabolite cortisone		NM_001124218.1			down
*il1β1* (S)	interleukin 1β1	endogenous pyrogen	Cytokines	NM_001124347.2	up	up	
*il1β2* (S)	interleukin 1β2	endogenous pyrogen		XM_021622166.1	up	up	
*il1β2* (K)	interleukin 1β2	endogenous pyrogen		XM_021622166.1		up	
*il1β3* (S)	interleukin 1β3	endogenous pyrogen		XM_021590496.1/AJ557021.2		up	
*tnf*α*2* (S)	tumor necrosis factor α2	potent pyrogen by stimulation of interleukin-1		NM_001124374.1	up		
*tnf*α*3* (S)	tumor necrosis factor α3	potent pyrogen by stimulation of interleukin-1		XM_021559781.1		up	
*il6* (S)	interleukin 6	stimulate lymphocyte and monocyte differentiation		NM_001124657.1		up	
*il8* (S)	interleukin 8	response to an inflammatory stimulus		NM_001124362.1	up		
*il11* (K)	interleukin 11	stimulate proliferation of hematopoietic stem cells and megakaryocyte progenitor cells		NM_001124382.1/AJ535687	up	up	
*sod3* (K)	extracellular superoxide dismutase (Cu-Zn)	convert superoxide radicals into hydrogen peroxide and oxygen		XM_021619043.1			down
*cat* (B)	catalase	protect cells from the toxic effects of hydrogen peroxide		XM_021564294.1			down
*c3-1a* (S)	Complement C3-1A	activation of the complement system	Complements	XM_021561545.2	up		
*c3-1b1* (K)	Complement C3-1B1	activation of the complement system		XM_021561577.2	up	up	
*c3-1b1* (S)	Complement C3-1B1	activation of the complement system		XM_021561577.2	up		up
*c3-1b2* (S)	Complement C3-1B2	activation of the complement system		XM_021595453.2		up	
*c3-3* (S)	Complement C3-3	activation of the complement system		XM_021568201.2	up	up	
*c3-4* (B)	Complement C3-4	activation of the complement system		XM_021557344.2	down		down
*c4* (K) or *c4b*	Complement C4	classical complement pathway		NM_001124385.1	up	up	
*c6* (S)	Complement C6	play a key role in the innate and adaptive immune response		NM_001124621.1		down	
*c7-1* (S) *or c7b*	Complement C7-1	play a key role in the innate and adaptive immune response		NM_001124618.1	up		up
*c7-2* (S) *or c7a*	Complement C7-2	play a key role in the innate and adaptive immune response		NM_001124407.1	down	down	
*c7-2* (K) *or c7a*	Complement C7-2	play a key role in the innate and adaptive immune response		NM_001124407.1	down	down	
*c7-2* (B) *or c7a*	Complement C7-2	play a key role in the innate and adaptive immune response		NM_001124407.1	down	down	down
*c8g* (S)	Complement component C8 gamma chain	regulate complement binding		NP_001117880.1		down	
*c8g* (B)	Complement component C8 gamma chain	regulate complement binding		NP_001117880.1			up
*cfb* (S)	Complement factor B	alternate pathway of the complement system		XM_036933232.1		up	
*bfc2b* (S)	Complement factor B/C2-B			NM_001124201		up	
*cfp1* (S)	Properdin	a positive regulator of the alternate pathway of complement		XM_021566443.2		up	
*c1qa* (S)	Complement C1q subcomponent subunit A	the first component of the serum complement system		XM_036968033.1	down		down
*cd93* (K)	Complement component C1q receptor	enhancement of phagocytosis in monocytes and macrophages		XM_021574853.2	down	down	
*mbl-h2* (K)	Mannan-binding lectin H2	calcium-dependent lectin involved in innate immune defense		NM_001160480.1	up	up	

In addition to upregulated cytokine genes (*il1β* subtypes, *tnfα* subtypes, and *il11*), ST and AT showed specifically upregulated *il8* and *il6*, respectively (**Figures 2** and **3**). IL6 and IL8 are two important proinflammatory cytokines that play an important role in regulating local or systemic inflammation (87). Studies showed both IL1α and IL1β subtypes could initiate the signal transduction and trigger the expression of IL6 and IL8 (12, 88). Consistently, this study revealed strong positive relationships between the expression of *il6*/*il8* and *il1β* subtypes (**Figures 2** and **3**, **Figures S2, S3, S6**). For example, the *il1β3* and *il6* were both upregulated in AT rather than ST (**Figures 3**). During evolution, the IL1α is evolving faster than IL1β, thus resulting in decreased sequence and functional homology between trout and mammalian IL1α orthologs (89, 90). Our further studies will investigate whether the evolutionally conserved IL1β exhibits subtype-specific IL6/IL8 expression regulation.

Compared to trout in ST, trout in AT exhibited more upregulated GO terms associated with immune defenses and the resulting intracellular signaling (**Figure 6** and **Table 2**), including GO:0051607, defense response to virus; GO:0035631, CD40 receptor complex; GO:0002768, immune response-regulating cell surface receptor signaling pathway; GO:2000353, positive regulation of endothelial cell apoptotic process; GO:0043123, positive regulation of I-κB kinase/NF-κB signaling; GO:0051092, positive regulation of NF-κB transcription factor activity; and GO:0042531, positive regulation of tyrosine phosphorylation of STAT protein. Despite limited studies on TNF-regulated intercellular and intracellular signaling transduction in teleosts, the *in vivo* studies on humans and rodents provide a potential model that could describe the immune mechanisms specifically activated in AT. Relevant to the GO terms of GO:2000353, GO:0043123, GO:0051092, and GO:0042531, previous biomedical studies showed TNFα activates the intracellular NF-κB signaling, while the cytoplasmic STAT serves as a negative regulator of TNFα-triggered NF-κB activation (91). The activation of NF-κB signaling and NF-κB transcriptional factors maintains an evolutionarily conserved and important role in initiating and coordinating the innate and adaptive immune responses (92). The phosphorylated STAT dimer will translate and localize to the nucleus, where it cannot interact with the TNFα-receptor complex. STAT localization to the nucleus allows a more robust TNFα-triggered NF-κB activation (91), enabling the trout to activate the immune defenses in response to *V. anguillarum* infection (**Figure 6E**).

**Table 2:** The enriched GO term lists.

GO Term	Function Description		Up- or Downregulation
**The enriched GO terms that are shared in comparisons of ST vs. CT and AT vs. CT**			
GO:0004883	glucocorticoid receptor activity	Molecular function, The stress response	Down
GO:0038050	glucocorticoid-activated sequence-specific DNA binding	Molecular function, The stress response	Down
GO:0031963	cortisol receptor activity	Molecular function, The stress response	Down
GO:1990794	basolateral part of the cell	Cellular component	Down
GO:0005496	steroid-binding	Molecular function, The stress response	Down
GO:0045944	positive regulation of transcription by RNA polymerase II	Biological process, Transcription	Down
GO:0051414	response to cortisol	Biological process, The stress response	Down
GO:0098531	direct ligand regulated sequence-specific DNA binding	Molecular function, Transcription	Down
GO:0042832	defense response to protozoan	Biological process, Immunomodulation	Up
GO:0032735	positive regulation of interleukin-12 production	Biological process, Immunomodulation	Up
GO:0043491	protein kinase B signaling	Biological process, Immunomodulation	Up
GO:0030890	positive regulation of B cell proliferation	Biological process, Immunomodulation	Up
**The enriched GO terms that are specifically identified in ST vs. CT**			
GO:0046688	response to copper ion	Biological process	Down
GO:0071383	cellular response to steroid hormone stimulus	Biological process, The stress response	Down
GO:0010628	positive regulation of gene expression	Biological process, The stress response	Down
GO:0006955	immune response	Biological process, Immunomodulation	Up
GO:0031625	ubiquitin-protein ligase binding	Molecular function, Immunomodulation	Up
GO:0005164	tumor necrosis factor receptor binding	Molecular function, Immunomodulation	Up
**The enriched GO terms that are specifically identified in AT vs. CT**			
GO:0005737	cytoplasm	Cellular component	Down
GO:0001046	core promoter sequence-specific DNA binding	Molecular function, Transcription	Down
GO:0003700	DNA-binding transcription factor activity	Molecular function, Transcription	Down
GO:1990239	steroid hormone binding	Molecular function, The stress response	Down
GO:0006325	chromatin organization	Biological process, Transcription	Down
GO:0051607	defense response to viruses	Biological process, Immunomodulation	Up
GO:0090037	positive regulation of protein kinase C signaling	Biological process, Signaling	Up
GO:0048304	positive regulation of isotype switching to IgG isotypes	Biological process, Immunomodulation	Up
GO:0035631	CD40 receptor complex	Cellular component, Immunomodulation	Up
GO:0002768	immune response-regulating cell surface receptor signaling pathway	Biological process, Immunomodulation	Up
GO:2000353	positive regulation of endothelial cell apoptotic process	Biological process, Immunomodulation	Up
GO:0051023	regulation of immunoglobulin secretion	Biological process, Immunomodulation	Up
GO:0051092	positive regulation of NF-κB transcription factor activity	Biological process, Immunomodulation	Up
GO:0043123	positive regulation of I-κB kinase/NF-κB signaling	Biological process, Immunomodulation	Up
GO:0043536	positive regulation of blood vessel endothelial cell migration	Biological process	Up
GO:0042531	positive regulation of tyrosine phosphorylation of STAT protein	Biological process, Immunomodulation	Up
GO:0042113	B cell activation	Biological process, Immunomodulation	Up
GO:0009897	external side of plasma membrane	Cellular component	Up
GO:0043547	positive regulation of GTPase activity	Biological process	Up

### Glucocorticoid Receptor and Mineralocorticoid Receptor

In addition to the GR, the teleost MR also serves as a receptor for stress perception. Our results showed the asymptomatic trout showed upregulated kidney *mrα* and *mrβ* expression. Consistently, previous studies showed MR and/or GR are expressed in immune tissues and regulate the immunomodulation (93–95). Moreover, increased stress hormone levels are observed in trout and zebrafish treated with the *V. anguillarum* vaccine (1, 96). Indeed, bidirectional communication exists between stress and immune responses, and low levels of stress (eustress) may result in enhanced immune competence (97). The slightly upregulated *mrα* and *mrβ* could act as an alarm and stimulate the immune system to fight against *V. anguillarum* infection, consistent with previous studies (10, 11).

Studies on humans, rodents, and other mammals showed that cytokines could affect the genes associated with the stress response through cytokine-specific mechanisms. For example, IL1 and IL6 exhibit positive effects, while the TNFα exhibits the opponent manners (16, 98, 99). These cytokines have also been reported to dysregulate and/or block the functions of GR subtypes (17). In teleosts, the immune responses regulated by the interactions between the genes in the stress response and cytokine networks are not homogeneous. Previous studies showed it is greatly affected by specific characteristics of challenges (environmental stressors or disease pathogens), target tissues (*in vitro* or *in vivo*; peripheral tissues or mucosal surfaces), and the adaptive life story of each species (bream, bass, or trout) (1, 5, 13, 100). In this study, the results showed that downregulated *mr* and *gr* subtypes exhibited strong negative relationships with cytokine genes of *il1β* and *tnfα* subtypes in AT and ST (**Figures 2** and **3**), which were partially consistent with the results in mammalian studies. Previous studies in sea bream showed that the stress response can suppress the gene expression of cytokines (13). These results indicated that the stress response and cytokine networks are intimately and bidirectionally linked, enabling teleosts to cope with challengers from environmental stimuli and/or pathogen invasion (8, 10, 83, 97).

After *V. anguillarum* infection, ST and AT both exhibited significantly downregulated GO terms associated with functions of *grα* and *grβ* (GO:0004883, glucocorticoid receptor activity; GO:0031963, cortisol receptor activity; GO:0005496, steroid binding), and significantly upregulated GO terms that are involved in *tnfα*-regulated immune responses (GO:0042832, defense response to protozoan; GO:0030890, positive regulation of B cell proliferation) (**Figure 6** and **Table 2**). The upregulated *il1β*, *il6*, *il8*, and *tnfα* genes are markers of M1 macrophage polarization, which activates the proinflammatory cytokine cascade against the pathogen invasion (101). The M1 macrophage-triggered proinflammatory cytokine cascade is suppressed by glucocorticoids and GR in basal conditions, but is activated by downregulated glucocorticoids and GR in an active infection (102). In this study, both ST and AT exhibited downregulated GO terms associated with cortisol and cortisol receptor functions and upregulated M1 macrophage polarization markers. These results suggest that activation of the proinflammatory cytokine cascade by M1 macrophage polarization is a general response for trout to fight pathogen invasion.

Compared to trout of AT, four KEGG pathways involved in steroid hormone biosynthesis and functions were downregulated in ST (**Figure 7**). Steroid hormones, which include corticosteroids and sex steroids, play an important role in regulating homeostasis *via* modulating metabolism, immunomodulation, salt balance, water balance, and reproduction. The KEGG analysis revealed that the genes associated with the biosynthesis of corticosteroids and sex steroids (ko04913 and ko00140) were significantly downregulated, suggesting that *V. anguillarum* infection severely dysregulated the homeostasis of the steroid hormone network in trout of ST (**Figure 7E**). The dyshomeostasis of steroid hormone might disturb the bidirectional link between stress and immune responses. Thus, steroid hormone receptors (such as kidney *mr* subtypes) might fail to transduce the “alarm” of pathogen infection to immune systems in symptomatic trout. Based on previous studies, the well-orchestrated stress response can be divided into three phases: alarm, resistance, and exhaustion (103–105). Downregulated steroid hormone biosynthesis might indicate that the ST was in an exhaustion phase, which is consistent with the human study showing death may be associated with an exhausted adrenal cortex (106–108).

Previously published chapters in the book of Fish Physiology (Biology of Stress in Fish, Volume 35) showed that, with the perception of health challenges, the induction of neuroendocrine cascades serves as the primary responses. The secondary response to stressors includes the physiological adjustments of hydromineral balance and immune function (3, 7, 109). Hydromineral dysfunction is a typical stress response because the altered adrenaline, which is induced by stressors, can change the gill blood flow and gill permeability and dysregulate the cardiovascular and respiratory functions (7, 109, 110). Consistently, our studies showed significantly downregulated KEGG pathways associated with aldosterone-regulated salt and water balance (ko04960 and ko04978) (**Figure 7E**), indicating that ST trout show hydromineral dyshomeostasis. Previous studies in Chinook salmon (*Oncorhynchus tshawytscha*) showed the hydromineral balance is changed during euthanasia (111), which is consistent with our KEGG results. Based on this data, we propose positive feedback between severe infection and imminent death: (1) infection and its resulting stress response disturb the hydromineral homeostasis, thus resulting in a moribund condition. (2) The moribund condition further exacerbated the dyshomeostasis of hydromineral functions, leading to death.

## Conclusions

Based on pairwise comparisons of CT, AT, and ST, we found the CT, AT, and ST show distinct transcriptional profiles of genes in stress and immune networks (**Figure 8**). The AT exhibited the eustress response, and eustress can stimulate the immune system to fight against bacterial infection. The ST exhibited a strong stress response, and the distress resulted in a secondary stress response, thus exacerbating immune dysfunctions and hydromineral dyshomeostasis. Regarding the immunomodulation, analysis of gene expression and pathway enrichments showed activation of the proinflammatory cytokine is a general response of AT and ST in responses to *V. anguillarum* infection. Additionally, the specifically upregulated complement and coagulation cascades and TNF-associated immune defenses probably enable the AT to fight the inflammatory pathogenesis and the resulting bleeding.

## Data Availability Statement

The datasets presented in this study can be found in online repositories. The names of the repository/repositories and accession number(s) can be found in the article/**Supplementary Material**.

## Ethics Statement

The animal study was reviewed and approved by Ethics Committees of Ocean University of China (permit number: 20141201).

## Author Contributions

Conceptualization: H-SW, J-FL, M-ZZ, and Z-SH. Project administration: Y-RX, CZ, and H-KZ. Supervision: H-SW, J-FL, and Z-SH. Methodology: Y-RX, CZ, H-KZ, X-DY, and M-QL. Writing—original draft: H-SW, Z-SH, and JD. Writing—review and editing: H-SW, Z-SH, and JD. All authors contributed to the article and approved the submitted version.

## Funding

Research in the authors’ laboratories is supported by grants from blue granary science and technology innovation (2019YFD0901000) and major technology innovation & application projects in Shandong province-breeding of the excellent trout species for open-sea farming (SD2019YY006).

## Conflict of Interest

The authors declare that the research was conducted in the absence of any commercial or financial relationships that could be construed as a potential conflict of interest.

## Abbreviations

CT, control trout; AT, asymptomatic trout; ST, symptomatic trout; DEGs, differential expression genes; GO, gene ontology; GR, glucocorticoid receptor; GH-IGF, growth hormone-insulin-like growth factor; KEGG, Kyoto Encyclopedia of Genes and Genomes; MR, mineralocorticoid receptor; PCA, principal component analysis; *V. anguillarum, Vibrio anguillarum.*
