# Supplementary material for: Transcriptional Profiles of Genes Related to Stress and Immune Response in Rainbow Trout (Oncorhynchus mykiss) Symptomatically or Asymptomatically Infected With Vibrio anguillarum
Source: Front Immunol. 2021 Apr 21;12:639489. doi: 10.3389/fimmu.2021.639489 (PMC8097155; doi:10.3389/fimmu.2021.639489)
Supplement: Supplementary file 1 [file DataSheet_1.docx]

**Figure S1 Hou et al. (2021)**

**
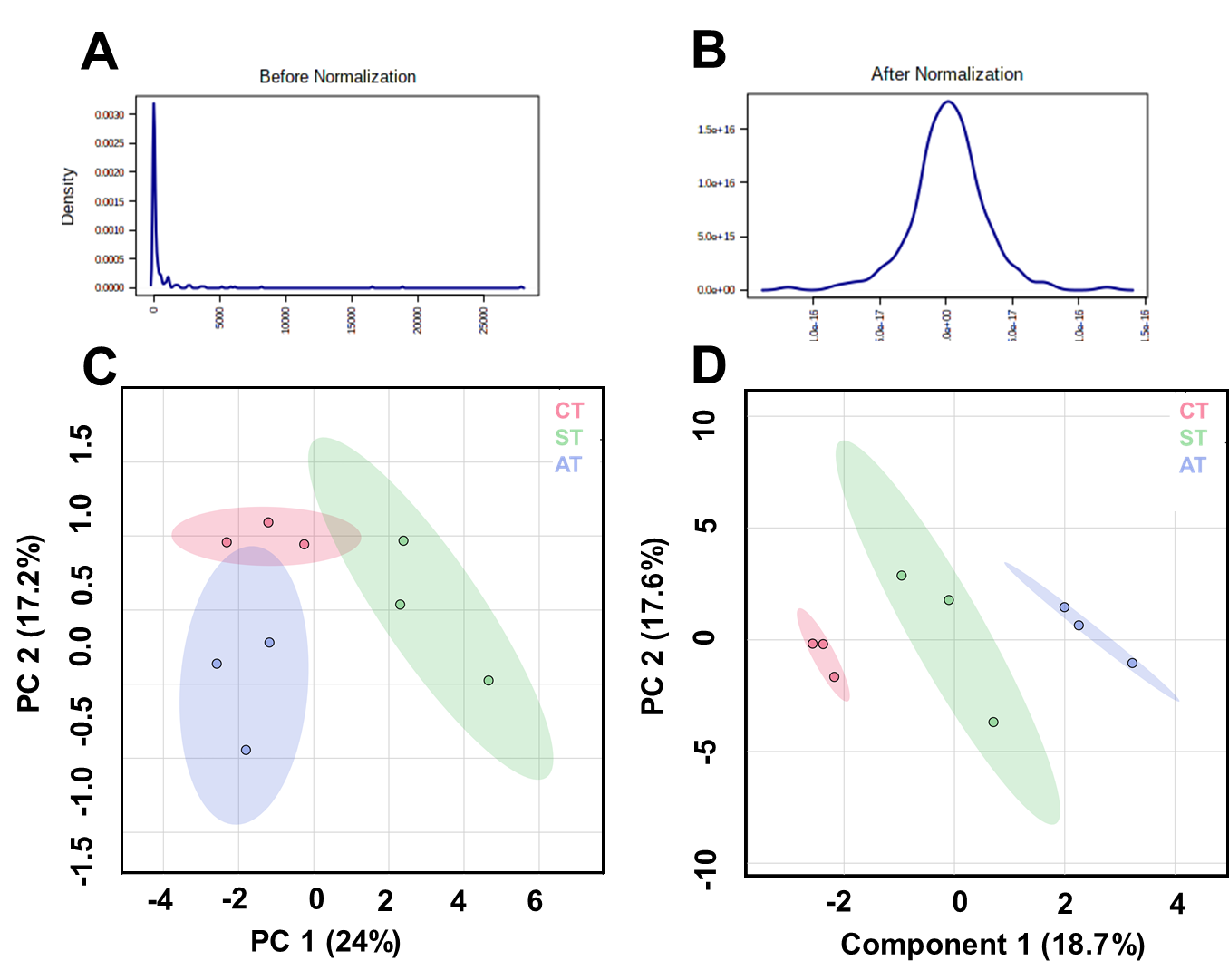
**

**Fig. S1***.* The original data of count (A) and the normalized count (B). The PCA (C) and PLS-DA (D) analyses of genes of stressful responses, cytokine and cellular functions, and complement systems.

After the normalization, the belt data distribution (Gaussian Distribution) is observed (A & B).

Separated PCA and PLS-DA plots were observed, indicating that the *V. anguillarum* infection resulted in significant alternations in genes of stressful responses, cytokine and cellular functions, and complement systems (C & D).

Figures and details of **Fig. S2 - S4 are shown in Excel (high resolution).**

**Fig. S2.** **Correlations of genes of stressful responses, cytokine and cellular functions, and complement systems between ST vs CT.**

K: kidney; S: Spleen; B: Brain. Abbreviations of genes are shown in the Table 1.

**Fig. S3.** **Correlations of genes of stressful responses, cytokine and cellular functions, and complement systems between AT vs CT.**

K: kidney; S: Spleen; B: Brain. Abbreviations of genes are shown in the Table 1.

**Fig. S4.** **Correlations of genes of stressful responses, cytokine and cellular functions, and complement systems between ST vs AT.**

K: kidney; S: Spleen; B: Brain. Abbreviations of genes are shown in the Table 1.

**Figure S5 Hou et al. (2021)**

**
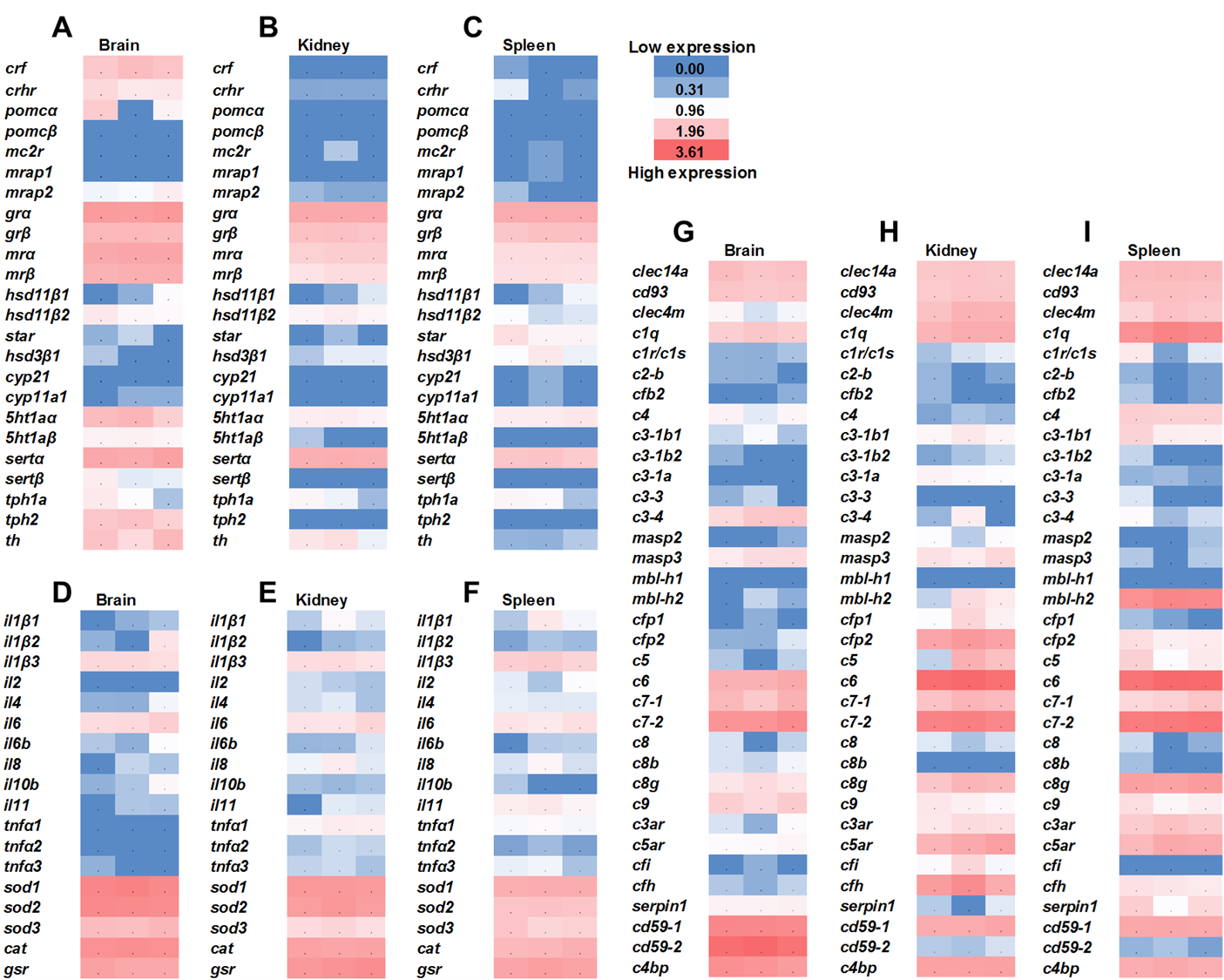
**

**Fig. S5.** **Basal expressions of genes of stressful responses (A - C), cytokine and cellular functions (D - F), and complement systems (G - I)**.

**Figure S6 Hou et al. (2021)**

**
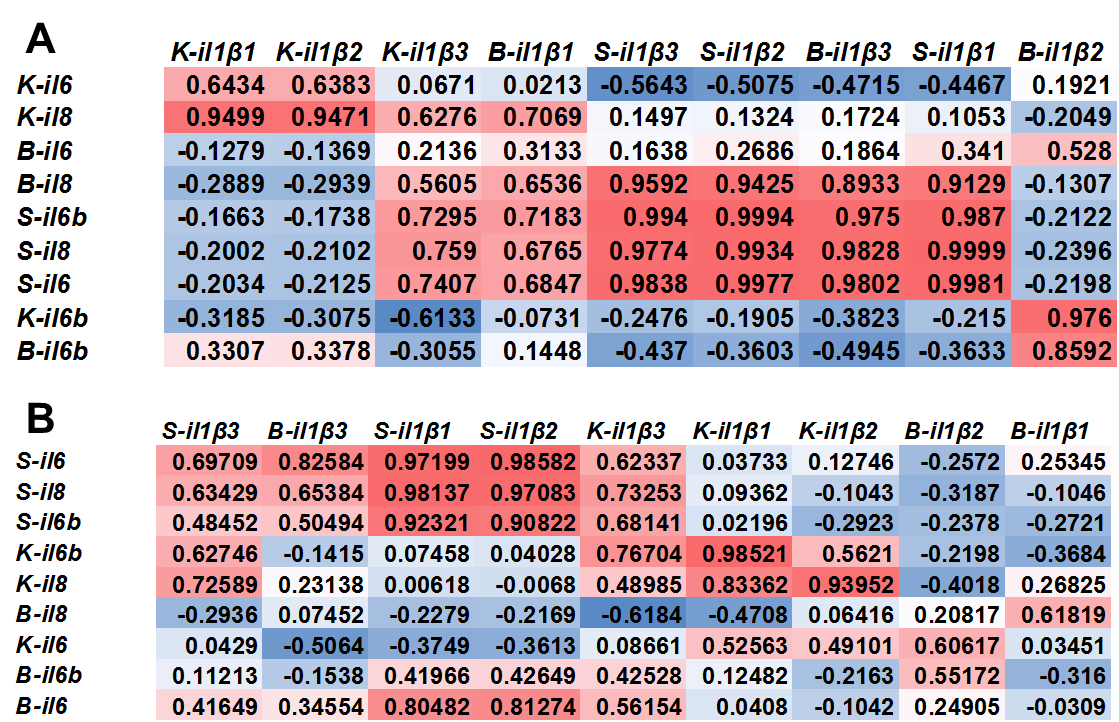
**

**Fig. S6.** **Correlations of genes of *il1β1*, *il1β2*, *il1β3*, *il6*, *il6b*, and *il8*.**

A: CT-ST; B: CT-AT.

K: kidney; S: Spleen; B: Brain. Abbreviations of genes are shown in the Table 1.

**Figure S7 Hou et al. (2021)**

1 2


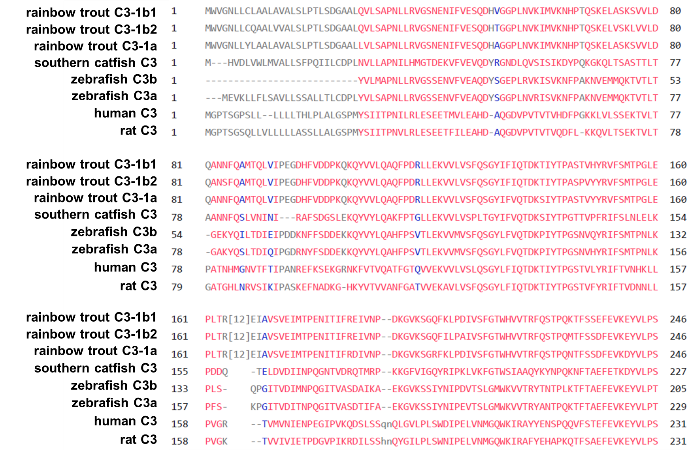

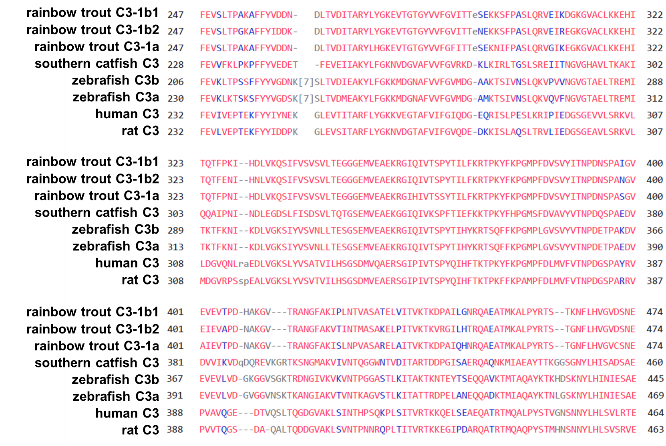


3 4


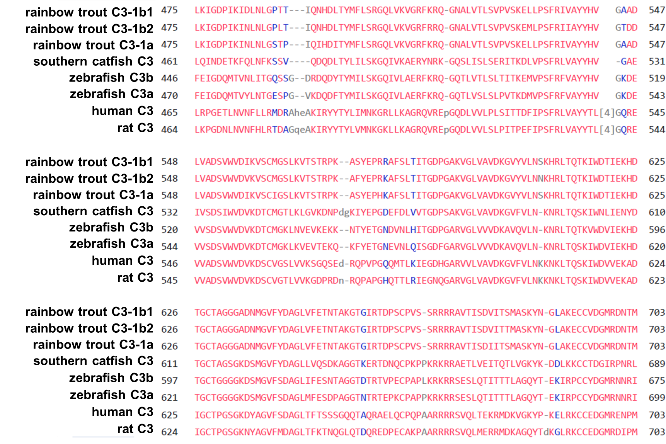

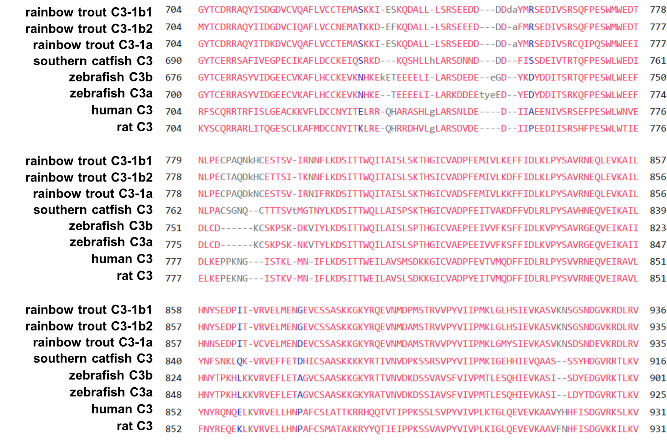


5 6


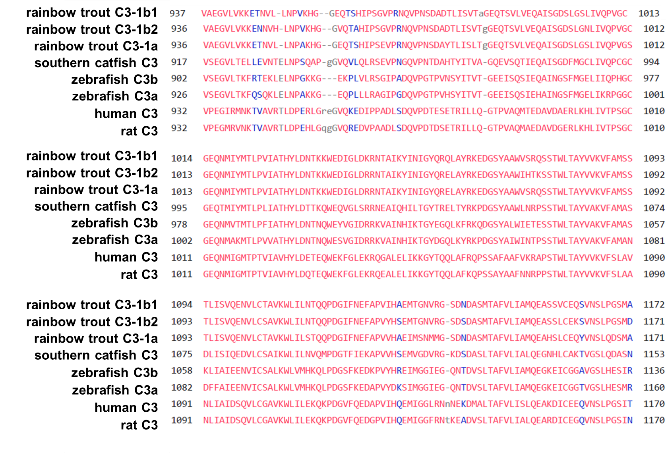

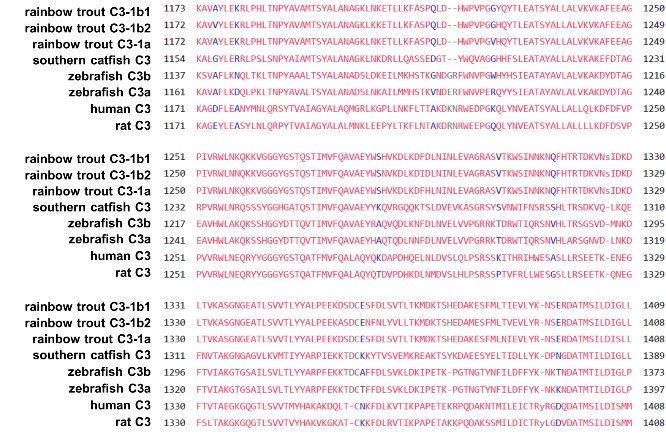


7


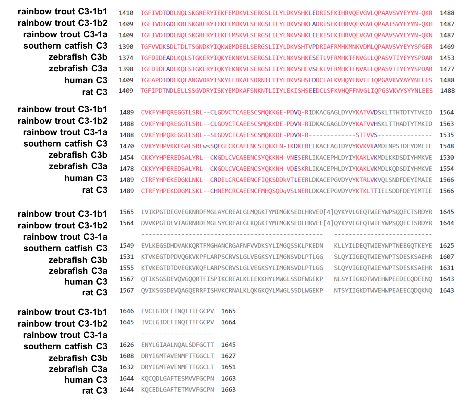


**Fig. S7 Alignment of mammalian and teleost C3.**

**Figure S8 Hou et al. (2021)**


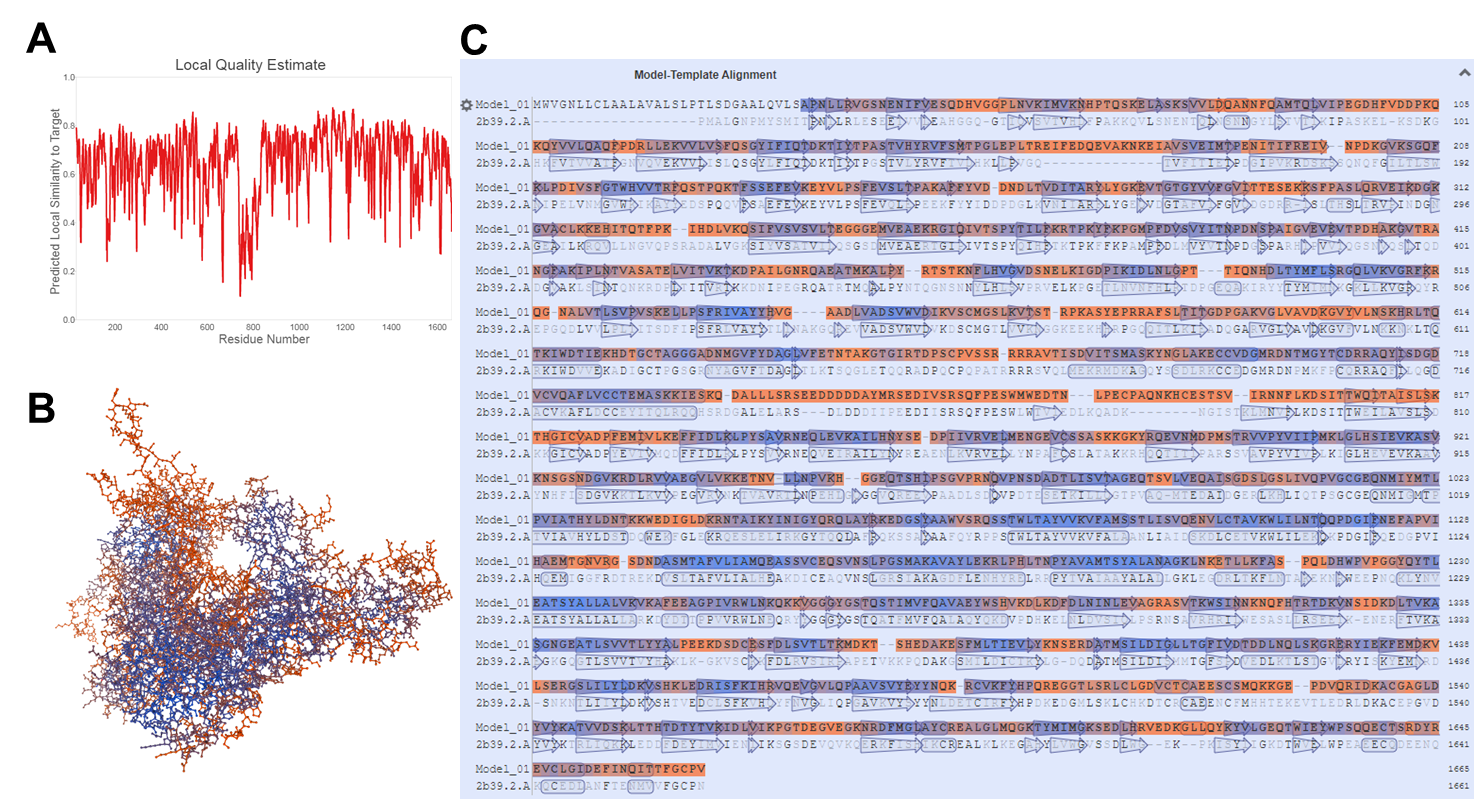


**Fig. S8.** **Parameters of SWISS-MODEL template of trout C3 and mammalian C3 (PDB ID: 2B39).**

Predicted local similarity (A), the ball and stick view (B) and of model-template alignment (C) of SWISS-MODEL template of trout C3 and mammalian C3. Blue shows the conserved motifs and red shows the less conserved motifs between model (mammalian C3, PDB ID: 2B39) and template (Trout C3).

**Figure S9 Hou et al. (2021)**


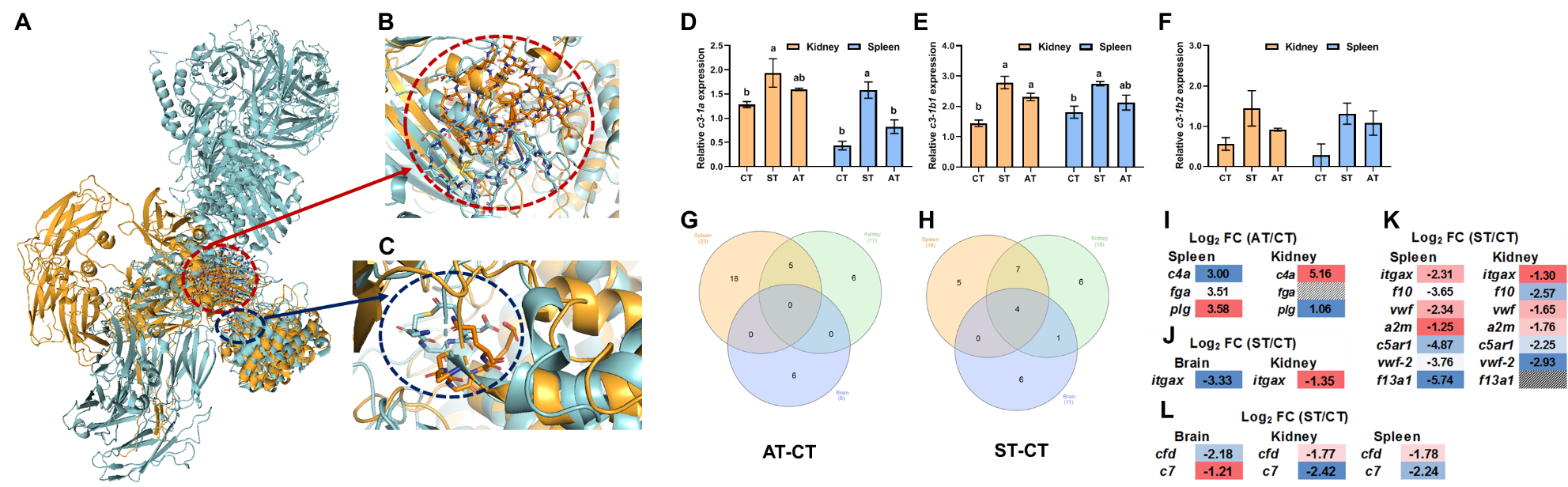


**Fig. S9. The 3D cartoon for the comparison of mammalian and trout C3 and the gene expression and functional analyses of Ko04610, complement and coagulation cascades**.

The 3D cartoon for the comparison of mammalian and trout C3 (A, whole structure). The ANATO (B) and thioester (GCGEQ sequences, C) domains are highlight.

D, E & F: Two-way ANOVA analyses of trout *c3* subtypes in kidney and spleen of CT, ST, and AT. Data are presented as mean ± SEM. The different letters indicate significant differences among CT, ST, and AT within the same tissue (p < 0.05, two-way ANOVA, followed by Tukey's Multiple Range test).

G and H: The Venn diagram of DEGs in Ko04610 (G: AT vs CT) and the Venn diagram of DEGs in Ko04610 (H: ST vs CT).

I, J, K and L: Heatmaps of overlapped DEGs in Venn diagram: I (overlapped DEGs between spleen and kidney in Figure G), J (overlapped DEGs between kidney and brain in Figure H), K (overlapped DEGs between spleen and kidney in Figure H) and L (overlapped DEGs between brain, spleen and kidney in Figure H). FC: Fold Change.

**Figure S10 Hou et al. (2021)**


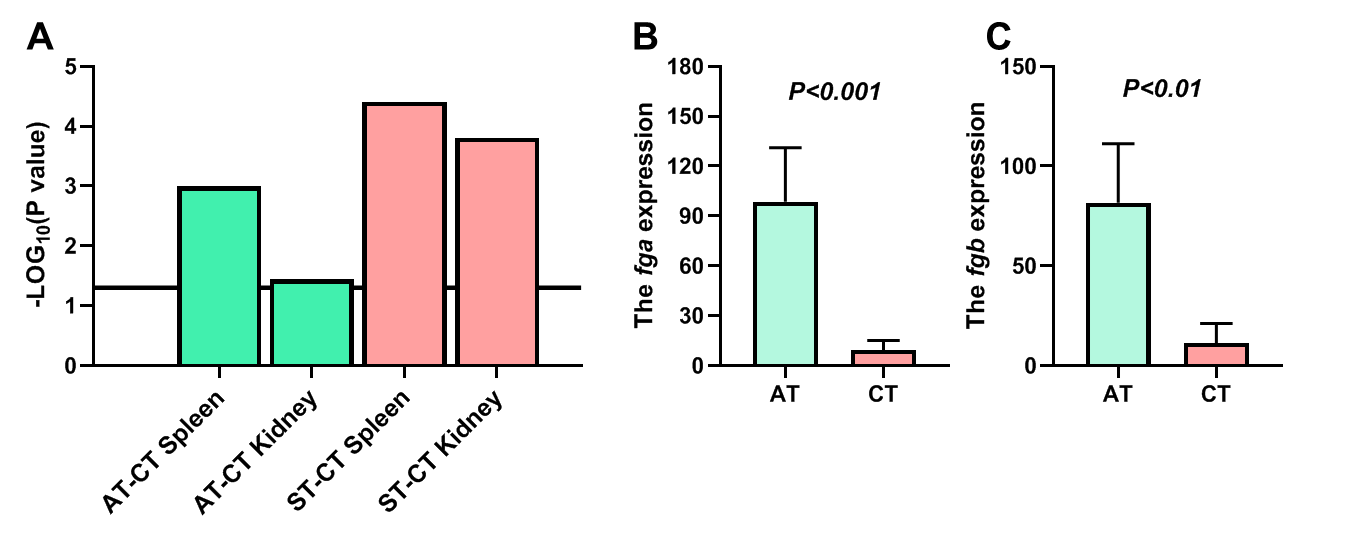


**Fig. S10.** Down-regulated platelet activation (ko04611) in AT and ST (A); *fga* and *fgb* expression between AT and CT in spleen (B & C).

**Table S1** Details of loading plot between ST and CT.

|  | PC1 | PC2 | PC3 | PC4 | PC5 | PC6 |
| --- | --- | --- | --- | --- | --- | --- |
| B-crf | -0.01691 | -0.00732 | -0.0053 | 0.045403 | -0.02869 | -0.50809 |
| B-crhr | 0.007493 | -0.0507 | 0.045323 | -0.02014 | -0.01063 | -0.22577 |
| B-pomcα | -0.0272 | -0.09495 | 0.17514 | -0.08569 | 0.049042 | 0.051392 |
| B-pomcβ | 0.11068 | -0.00697 | 0.015912 | -0.11677 | 0.054901 | -0.65994 |
| B-mc2r | 0.046635 | 0.028051 | 0.023821 | -0.04449 | 0.019325 | -0.41457 |
| B-mrap1 | -0.01296 | -0.07313 | -0.00171 | -0.00493 | 0.002099 | -0.00547 |
| B-mrap2 | -0.00683 | -0.00511 | 0.005074 | 0.011556 | 0.062728 | -0.01108 |
| B-grα | -0.02084 | 0.001385 | 0.013402 | -0.01319 | 0.013164 | -0.00311 |
| B-grβ | 0.003032 | -0.00225 | 0.012944 | -0.01429 | -0.00571 | 0.002195 |
| B-mrα | -0.01267 | 0.041578 | 0.003708 | -0.04677 | 0.012694 | 0.001858 |
| B-mrβ | -0.01721 | 0.024473 | -0.00515 | -0.05692 | 0.02297 | 0.011133 |
| B-hsd11β1 | 0.075488 | -0.01966 | -0.06727 | 0.058483 | 0.2202 | 0.005163 |
| B-hsd11β2 | -0.06535 | 0.080532 | 0.055183 | -0.00465 | -0.04288 | 0.011709 |
| B-star | 0.001774 | 0.067347 | -0.01665 | -0.02562 | -0.1816 | 0.031201 |
| B-hsd3β1 | 0.040296 | -0.06447 | 0.15502 | -0.03294 | -0.07289 | 0.050134 |
| B-cyp21 | -0.01299 | -0.07317 | -0.00171 | -0.00496 | 0.002105 | 0.012285 |
| B-cyp11a1 p450scc | -0.01578 | -0.0936 | -0.12643 | -0.0909 | 0.088337 | -0.03457 |
| B-5ht1aα | -0.01408 | -0.0624 | -0.01092 | -0.01233 | -0.07527 | -0.01751 |
| B-5ht1aβ | -0.02181 | -0.0218 | 0.011986 | 0.021402 | -0.0217 | -0.00913 |
| B-sertα | -0.02124 | -0.02026 | 0.008372 | -0.01309 | 0.030476 | 0.002541 |
| B-sertβ | -0.01195 | -0.02411 | 0.081293 | 0.015299 | -0.04098 | -0.00668 |
| B-tph1a | -0.00143 | -0.02567 | 0.076088 | 0.022308 | -0.15366 | 0.013767 |
| B-tph2 | -0.02282 | -0.03972 | -0.00736 | -0.00631 | -0.04527 | -0.0133 |
| B-th | -0.01992 | -0.0436 | 0.056083 | 0.024616 | 0.069048 | -0.01972 |
| B-il1β1 | 0.040871 | -0.04188 | -0.06086 | 0.12464 | 0.078602 | -0.01174 |
| B-il1β2 | -0.02354 | -0.03312 | 0.073592 | 0.030991 | 0.30073 | -0.03335 |
| B-il1β3 | 0.002504 | 5.45E-05 | 0.021147 | 0.008106 | -0.02077 | -0.00302 |
| B-il2 | 0.031845 | -0.09279 | 0.023684 | 0.075139 | -0.01719 | 0.028043 |
| B-il4 | 0.050711 | -0.01541 | 0.005756 | -0.04381 | 0.16892 | 0.018966 |
| B-il6 | -0.00753 | -0.00174 | 0.002997 | -0.01039 | 0.043449 | 0.004338 |
| B-il6b | -0.02672 | -0.03892 | 0.027822 | -0.02199 | 0.13606 | 0.008882 |
| B-il8 | 0.01384 | 0.06359 | -0.04129 | 0.20595 | -0.03199 | 0.01763 |
| B-il10b | 0.040812 | -0.01104 | -0.01035 | 0.066222 | 0.13678 | 0.01606 |
| B-il11 | 0.05585 | -0.04655 | -0.08387 | -0.02203 | 0.073549 | -0.02887 |
| B-tnfα1 | 0.073086 | 0.040049 | 0.044836 | 0.008671 | 0.004798 | 0.013716 |
| B-tnfα2 | 0.086101 | 0.046001 | 0.054778 | 0.033049 | -0.00175 | 0.010704 |
| B-tnfα3 | 0.031482 | -0.05708 | 0.13208 | 0.09642 | -0.08593 | -0.00975 |
| B-sod1 | -0.02853 | -0.01484 | -0.00556 | -0.0131 | -0.019 | -0.00408 |
| B-sod2 | -0.0287 | -0.0163 | 0.008852 | -0.01534 | -0.01126 | -0.00565 |
| B-sod3 | -0.03192 | 0.018737 | 0.015946 | -0.00645 | 0.020394 | -0.0023 |
| B-cat | -0.03553 | 0.034465 | 0.006962 | -0.00808 | -0.00788 | -0.00997 |
| B-gsr | -0.01954 | -0.01475 | 0.031005 | 0.017881 | -0.01949 | 0.00226 |
| B-clec14a | -0.01717 | -0.02196 | 0.027265 | -0.01282 | 0.000493 | -0.01365 |
| B-cd93 | -0.04216 | -0.01435 | 0.017398 | 0.006713 | -0.0072 | -0.01464 |
| B-clec4m | 0.061948 | 0.011801 | 0.092683 | 0.015277 | 0.005069 | 0.003134 |
| B-c1q | 0.009514 | -0.01343 | 0.008665 | 0.06196 | -0.02802 | 0.007485 |
| B-c1r/c1s | -0.03351 | 0.074872 | 0.011877 | -0.04627 | 0.053111 | 0.010885 |
| B-c2-b | 0.053483 | -0.06707 | 0.024654 | -0.03219 | -0.10915 | -0.01463 |
| B-cfb2 | -0.02278 | 0.021642 | -0.01271 | -0.07101 | 0.14989 | 0.002987 |
| B-c4 | 0.001506 | 0.024495 | 0.084833 | 0.055155 | 0.008668 | 0.002795 |
| B-c3-1b1 | 0.10734 | 0.017738 | 0.004585 | -0.01946 | -0.05544 | -0.00807 |
| B-c3-1b2 | 0.041905 | 0.050084 | 0.099955 | -0.20243 | 0.003095 | 0.03601 |
| B-c3-1a | 0.029733 | -0.07169 | 0.02694 | 0.073318 | -0.01793 | 0.002298 |
| B-c3-3 | 0.014528 | -0.12548 | -0.06626 | -0.08984 | -0.15164 | -0.01533 |
| B-c3-4 | -0.07135 | 0.051675 | -0.04923 | -0.05277 | 0.044114 | -0.02264 |
| B-masp2 | -0.04125 | -0.06025 | -0.0074 | 0.043456 | 0.11464 | -0.01464 |
| B-masp3 | -0.00376 | -0.03754 | -0.02392 | 0.033984 | 0.016913 | -0.00869 |
| B-mbl-h2 | 0.050885 | -0.05198 | -0.1396 | -0.00325 | 0.020161 | 0.049041 |
| B-cfp1 | -0.03118 | -0.05609 | -0.09175 | 0.008639 | -0.08029 | -0.0101 |
| B-cfp2 | -0.07151 | -0.0743 | 0.010935 | 0.14872 | 0.062062 | -0.03765 |
| B-c5 | -0.01847 | -0.0291 | 0.11814 | -0.08157 | 0.15364 | 0.037211 |
| B-c6 | -0.01438 | -0.00469 | 0.020851 | 0.038768 | 0.01179 | -0.00455 |
| B-c7-1 | -0.00932 | -0.0121 | 0.047891 | 0.006372 | 0.049662 | -0.00396 |
| B-c7-2 | -0.05816 | 0.008703 | 0.005764 | 0.007635 | 0.01872 | -0.01771 |
| B-c8 | -0.0155 | -0.07023 | 0.17692 | 0.034501 | 0.08971 | 0.002273 |
| B-c8b | 0.005325 | -0.06364 | 0.012644 | -0.03458 | 0.063332 | 0.006529 |
| B-c8g | 0.0092 | -0.01668 | -0.00429 | 0.022258 | -0.02222 | -0.00228 |
| B-c9 | -0.0294 | -0.01705 | 0.021835 | -0.02882 | 0.037995 | 0.00675 |
| B-c3ar | 0.014894 | -0.02499 | 0.1179 | 0.13705 | 0.07171 | 0.011554 |
| B-c5ar | 0.006517 | -0.01934 | 0.011802 | 0.010134 | 0.016843 | 0.009393 |
| B-cfi | -0.00346 | -0.0683 | -0.11036 | -0.09719 | -0.0389 | 0.044657 |
| B-cfh | 0.004709 | -0.05791 | 0.044848 | 0.003611 | 0.055045 | 0.012569 |
| B-serpin1 | -0.04061 | -0.05642 | 0.002518 | 0.018905 | -0.00082 | -0.01943 |
| B-cd59-1 | -0.023 | -0.00251 | 0.009148 | -0.02302 | -0.00991 | 0.000978 |
| B-cd59-2 | -0.03571 | -0.00248 | -0.0043 | -0.01589 | -0.02709 | -0.00085 |
| B-c4bp | -0.03134 | -0.01077 | 0.017588 | -0.0062 | -0.03891 | -0.0147 |
| K-crhr | -0.09306 | 0.02618 | 0.000212 | 0.016182 | -0.02169 | -0.02339 |
| K-pomcβ | 0.052493 | 0.031757 | 0.020144 | -0.07003 | 0.028608 | 0.008656 |
| K-mc2r | -0.00711 | 0.038676 | -0.10076 | 0.087243 | -0.14949 | -0.00218 |
| K-mrap2 | 0.015665 | -0.07133 | 0.042545 | -0.01165 | -0.01709 | -0.00683 |
| K-grα | -0.03141 | 0.008061 | 0.004157 | -0.02525 | 0.006065 | -0.00291 |
| K-grβ | -0.02648 | 0.047789 | 0.014788 | -0.02702 | -0.01717 | 0.000823 |
| K-mrα | -0.07508 | 0.056261 | 0.007763 | 0.043014 | -0.02299 | -0.00354 |
| K-mrβ | -0.07143 | 0.019529 | -0.02038 | -0.00085 | 0.001451 | -0.00134 |
| K-hsd11β1 | 0.018512 | -0.01472 | -0.08723 | 0.021031 | 0.20782 | 0.048369 |
| K-hsd11β2 | -0.09576 | -0.01088 | -0.01133 | 0.076278 | -0.05884 | -0.03229 |
| K-star | 0.21788 | 0.076389 | -0.02929 | -0.03373 | -0.04736 | 0.058422 |
| K-hsd3β1 | 0.11592 | -0.00188 | -0.01002 | 0.030432 | 0.042471 | 0.020241 |
| K-cyp21 | 0.021349 | 0.012057 | 0.039834 | 0.066049 | -0.02084 | -0.004 |
| K-cyp11a1 p450scc | 0.061448 | -0.03911 | 0.055143 | 0.13446 | -0.03537 | -0.01685 |
| K-5ht1aα | -0.03728 | -0.07078 | -0.00837 | 0.003667 | -0.02509 | -0.01539 |
| K-5ht1aβ | -0.04818 | -0.0026 | 0.091324 | -0.04397 | -0.05325 | -0.002 |
| K-sertα | -0.01959 | -0.01352 | 0.000807 | -0.01256 | -0.00508 | -0.00243 |
| K-tph1a | 0.055237 | -0.06403 | 0.057838 | -0.04192 | -0.12102 | 0.019678 |
| K-th | 0.12961 | -0.03607 | 0.021051 | -0.04827 | -0.10456 | -0.019 |
| K-il1β1 | 0.090907 | -0.17569 | -0.09001 | -0.01833 | 0.016298 | 0.054398 |
| K-il1β2 | 0.10016 | -0.20313 | -0.09302 | 0.022454 | 0.10621 | -0.00375 |
| K-il1β3 | 0.018283 | -0.02079 | 0.012578 | -0.01039 | -0.0285 | 0.004487 |
| K-il2 | 0.013351 | 0.095836 | 0.051155 | -0.09069 | -0.01697 | -0.00661 |
| K-il4 | 0.034731 | -0.07901 | -0.00975 | -0.04172 | -0.05607 | 0.002145 |
| K-il6 | -0.02917 | -0.05296 | -0.02638 | -0.10465 | 0.095503 | 0.045211 |
| K-il6b | -0.0443 | 0.020658 | 0.003402 | 0.027868 | 0.092197 | -0.02718 |
| K-il8 | 0.099099 | -0.12129 | -0.0307 | 0.053766 | -0.0691 | -0.03209 |
| K-il10b | 0.026 | -0.08221 | 0.062024 | 0.16009 | -0.02985 | -0.01999 |
| K-il11 | 0.15188 | -0.14722 | -0.06223 | 0.073679 | 0.057608 | -0.02881 |
| K-tnfα1 | -0.01289 | -0.05496 | -0.02738 | -0.00373 | 0.013031 | 0.008398 |
| K-tnfα2 | -0.04127 | -0.14348 | -0.07974 | -0.06522 | 0.03297 | -0.04509 |
| K-tnfα3 | 0.045013 | -0.08478 | -0.00636 | -0.03782 | -0.02588 | -0.01188 |
| K-sod1 | -0.03147 | 0.010851 | 0.013458 | -0.0126 | -0.01135 | -0.00854 |
| K-sod2 | -0.05665 | -0.02041 | -0.01934 | -0.00281 | -0.02696 | -0.01037 |
| K-sod3 | -0.08282 | 0.051121 | -0.01694 | -0.03165 | -0.03355 | -0.02323 |
| K-cat | -0.06304 | -0.01804 | 0.005779 | -0.01779 | 0.000191 | -0.0096 |
| K-gsr | 0.025717 | 0.026945 | 0.003241 | -0.02024 | 0.046294 | 0.005444 |
| K-clec14a | -0.05133 | -0.01145 | -0.01631 | -0.03376 | -0.00521 | -0.0026 |
| K-cd93 | -0.09 | 0.01281 | -0.03058 | -0.03656 | 0.002064 | 0.013332 |
| K-clec4m | -0.00503 | 0.10708 | -0.0201 | -0.04667 | 0.033642 | 0.004393 |
| K-c1q | -0.054 | -0.00187 | -0.05248 | -0.10648 | 0.046928 | 0.00279 |
| K-c1r/c1s | -0.00012 | -0.00955 | -0.01461 | 0.1169 | 0.01988 | -0.02083 |
| K-c2-b | 0.045377 | 0.078177 | 0.17141 | 0.014959 | 0.040303 | -0.0045 |
| K-cfb2 | 0.057004 | 0.051036 | 0.12757 | -0.04406 | -0.04018 | 0.015002 |
| K-c4 | 0.11954 | 0.16586 | -0.00513 | -0.07954 | 0.043897 | 0.008666 |
| K-c3-1b1 | 0.10956 | -0.11618 | -0.03004 | -0.0372 | 0.005701 | 0.019063 |
| K-c3-1b2 | 0.05667 | -0.15595 | -0.06328 | 0.047663 | 0.09491 | 0.007292 |
| K-c3-1a | 0.024196 | -0.10633 | 0.001798 | -0.07648 | 0.002995 | 0.030652 |
| K-c3-3 | 0.083898 | -0.02421 | 0.035163 | 0.010521 | 0.008937 | 0.002388 |
| K-c3-4 | 0.078043 | -0.00067 | -0.12086 | -0.0876 | -0.20802 | -0.03941 |
| K-masp2 | -0.08863 | 0.13224 | 0.044179 | -0.18762 | 0.10833 | -0.00373 |
| K-masp3 | -0.01443 | -0.03017 | 0.025694 | -0.01403 | 0.059892 | 0.010974 |
| K-mbl-h2 | 0.083116 | -0.05973 | -0.12947 | 0.051172 | 0.040773 | 0.008794 |
| K-cfp1 | -0.03933 | 0.037237 | -0.07824 | 0.097752 | -0.07596 | -0.02494 |
| K-cfp2 | -0.03308 | -0.08412 | -0.03856 | 0.008605 | -0.02177 | 0.016501 |
| K-c5 | 0.001321 | -0.11341 | -0.27825 | 0.12996 | 0.084896 | -0.01875 |
| K-c6 | -0.04098 | 0.023423 | -0.01218 | -0.05498 | -0.01223 | 0.004312 |
| K-c7-1 | 0.044938 | -0.15163 | -0.03982 | 0.031188 | 0.007487 | -0.03291 |
| K-c7-2 | -0.10146 | 0.039583 | -0.02069 | -0.0574 | -0.02452 | -0.00092 |
| K-c8 | 0.002799 | -0.15038 | 0.038179 | -0.0273 | 0.042474 | 0.055587 |
| K-c8b | 0.097437 | -0.06054 | 0.038785 | 0.043476 | 0.00165 | 0.005269 |
| K-c8g | 0.011294 | -0.07319 | -0.04295 | 0.026318 | 0.009845 | -0.00569 |
| K-c9 | 0.019132 | -0.02646 | 0.046003 | -0.01745 | -0.0416 | 0.014488 |
| K-c3ar | 0.008255 | 0.01532 | -0.02372 | -0.03815 | 0.024771 | -0.00057 |
| K-c5ar | -0.01627 | 0.040192 | -0.02209 | -0.02926 | 0.067584 | 0.000335 |
| K-cfi | -0.05994 | -0.12422 | -0.124 | -0.0302 | -0.09565 | -0.01385 |
| K-cfh | -0.04845 | -0.14759 | -0.06768 | -0.01819 | -0.09207 | 0.027232 |
| K-serpin1 | 0.052927 | 0.048903 | 0.1799 | 0.026107 | 0.17573 | 0.001138 |
| K-cd59-1 | -0.02433 | -0.03434 | -0.00254 | -0.01238 | 0.02379 | 0.003812 |
| K-cd59-2 | -0.06881 | -0.05676 | -0.00625 | -0.01386 | 0.052812 | -0.00344 |
| K-c4bp | -0.0205 | -0.00512 | 0.01145 | 0.00233 | -0.00071 | -0.00244 |
| S-crf | 0.00891 | -0.11645 | 0.042351 | -0.21949 | 0.019886 | 0.040082 |
| S-crhr | -0.10971 | -0.12398 | 0.20505 | -0.04212 | -0.03088 | -0.01652 |
| S-pomcα | -0.01299 | -0.07317 | -0.00171 | -0.00496 | 0.002105 | -0.00382 |
| S-mc2r | 0.002091 | 0.03329 | -0.09848 | -0.16619 | -0.01484 | 0.030747 |
| S-mrap1 | 0.009258 | -0.03833 | -0.10953 | -0.16002 | -0.01234 | 0.0058 |
| S-mrap2 | -0.00791 | -0.13038 | 0.14495 | 0.027426 | -0.09771 | -0.01002 |
| S-grα | -0.03803 | 0.013272 | 0.007886 | -0.00396 | -0.00764 | 0.001756 |
| S-grβ | -0.06835 | 0.024493 | -0.01769 | -0.01237 | 0.00236 | -0.01663 |
| S-mrα | -0.07364 | -0.04816 | -0.01052 | -0.00245 | -0.00706 | 0.027521 |
| S-mrβ | -0.08365 | -0.04323 | -0.01161 | 0.023715 | -0.02678 | 0.001294 |
| S-hsd11β1 | -0.02477 | -0.05612 | -0.1396 | 0.069305 | 0.20039 | -0.03719 |
| S-hsd11β2 | -0.06724 | 0.010199 | 0.064431 | 0.010975 | -0.02636 | -0.00968 |
| S-star | 0.00319 | 0.029884 | 0.071948 | -0.08726 | -0.01763 | 0.011567 |
| S-hsd3β1 | 0.094847 | 0.047542 | -0.02959 | -0.11041 | -0.0298 | 0.007912 |
| S-cyp21 | -0.02921 | -0.06268 | -0.08523 | 0.007962 | -0.07341 | 0.001791 |
| S-cyp11a1 p450scc | 0.072027 | -0.02618 | -0.06853 | 0.054613 | -0.09668 | 0.014455 |
| S-5ht1aα | 0.084491 | 0.036575 | 0.023656 | -0.07066 | 0.056829 | 0.008109 |
| S-5ht1aβ | 0.021351 | 0.012059 | 0.03985 | 0.066075 | -0.02085 | 0.004594 |
| S-sertα | 0.024054 | -0.02008 | 0.009831 | -0.02304 | -0.01764 | 1.54E-05 |
| S-tph1a | 0.052851 | 0.042775 | 0.043017 | -0.07446 | -0.13434 | 0.003221 |
| S-tph2 | -0.013 | -0.07318 | -0.00171 | -0.00496 | 0.002106 | 0.008187 |
| S-th | 0.035909 | -0.01818 | -0.01311 | -0.15598 | 0.12123 | -0.00108 |
| S-il1β1 | 0.26852 | 0.030371 | -0.06093 | 0.009893 | 0.029907 | 0.019519 |
| S-il1β2 | 0.26593 | 0.011765 | 0.024305 | 0.060669 | 0.036888 | 0.047615 |
| S-il1β3 | 0.022424 | 0.009842 | 0.027864 | 0.06136 | -0.05028 | -0.00998 |
| S-il2 | -0.03094 | -0.04777 | 0.088689 | 0.12265 | 0.040912 | -0.01823 |
| S-il4 | 0.001837 | -0.04441 | 0.022192 | 0.00891 | -0.00752 | -0.01264 |
| S-il6 | 0.10192 | 0.050589 | 0.077174 | 0.048984 | 0.025597 | 0.01779 |
| S-il6b | 0.19505 | 0.001121 | 0.001185 | 0.14725 | 0.047565 | 0.036768 |
| S-il8 | 0.23151 | 0.022956 | 0.015271 | -0.01094 | -0.01872 | -0.01785 |
| S-il10b | 0.064631 | -0.04938 | 0.10047 | -0.08348 | -0.02086 | -0.00531 |
| S-il11 | 0.19506 | 0.090615 | 0.056932 | -0.06432 | 0.008424 | -0.03184 |
| S-tnfα1 | 0.13789 | 0.031337 | 0.05733 | 0.071422 | -0.02455 | 0.015286 |
| S-tnfα2 | 0.21381 | -0.02363 | 0.02471 | 0.046977 | -0.03973 | -0.00705 |
| S-tnfα3 | 0.11489 | 0.015261 | 0.052909 | 0.012894 | -0.10347 | 0.014133 |
| S-sod1 | -0.00808 | 0.004661 | 0.002871 | -0.04171 | 0.015513 | -0.00195 |
| S-sod2 | -0.00944 | -0.02367 | -0.00681 | -0.02482 | -0.00197 | -0.0004 |
| S-sod3 | -0.07523 | -0.0253 | 0.059167 | 0.050586 | -0.0412 | -0.0229 |
| S-cat | -0.05537 | 0.019566 | -0.01195 | -0.03475 | 0.03535 | -0.00726 |
| S-gsr | 0.015228 | -0.00278 | -0.00153 | 0.001127 | -0.00813 | 0.001888 |
| S-clec14a | -0.01747 | 0.039144 | 0.014154 | -0.00029 | -0.01455 | -0.0018 |
| S-cd93 | -0.03529 | 0.034414 | 0.020392 | 0.015192 | -0.02076 | -0.0038 |
| S-clec4m | -0.067 | -0.02673 | -0.01468 | 0.199 | -0.06849 | -0.03397 |
| S-c1q | -0.05718 | 0.017651 | -0.02379 | 0.052623 | -0.02719 | -0.01342 |
| S-c1r/c1s | -0.02651 | -0.04813 | 0.2194 | -0.0251 | 0.054477 | 0.013485 |
| S-c2-b | -0.04491 | -0.13919 | 0.086158 | -0.12659 | 0.07746 | 0.039847 |
| S-cfb2 | -0.03059 | -0.17629 | 0.17215 | 0.08396 | -0.01516 | 0.025968 |
| S-c4 | -0.02908 | -0.04047 | 0.060123 | 0.21341 | -0.07838 | -0.02969 |
| S-c3-1b1 | 0.075019 | -0.0629 | 0.092089 | -0.05084 | -0.02622 | 0.005254 |
| S-c3-1b2 | 0.058508 | -0.12215 | 0.091894 | -0.05139 | -0.029 | 0.021334 |
| S-c3-1a | 0.1379 | -0.03624 | 0.025833 | -0.03791 | -0.04762 | 0.011487 |
| S-c3-3 | 0.079085 | -0.06407 | 0.095837 | -0.10632 | -0.01031 | 0.026135 |
| S-c3-4 | -0.02658 | -0.11709 | 0.11037 | 0.031327 | -0.00401 | 0.019408 |
| S-masp2 | -0.02247 | 0.032012 | 0.031037 | 0.14102 | 0.15346 | 0.009808 |
| S-masp3 | -0.02154 | -0.12994 | 0.095765 | 0.09489 | 0.045069 | 0.00641 |
| S-mbl-h2 | 0.05476 | 0.043863 | 0.006534 | -0.00822 | 0.014836 | 0.005321 |
| S-cfp1 | 0.078699 | -0.11863 | 0.08009 | -0.07503 | -0.13137 | -0.011 |
| S-cfp2 | -0.10405 | 0.026089 | 0.041294 | -0.02692 | -0.02073 | -0.00758 |
| S-c5 | -0.0655 | -0.02078 | 0.14232 | 0.06323 | -0.03697 | -0.01098 |
| S-c6 | -0.0031 | 0.055819 | -0.00707 | -0.01424 | 0.018661 | 0.009999 |
| S-c7-1 | 0.16527 | 0.039953 | 0.031867 | -0.03287 | 0.099503 | 0.045222 |
| S-c7-2 | -0.08243 | 0.062324 | 0.009825 | 0.030574 | -0.00356 | -0.02178 |
| S-c8 | 0.016182 | -0.0938 | 0.15648 | -0.07072 | 0.044795 | -0.02181 |
| S-c8b | -0.03542 | -0.1302 | 0.071636 | -0.03297 | -0.0488 | -0.03379 |
| S-c8g | -0.02452 | 0.019738 | 0.017337 | -0.01103 | 0.01647 | -0.01108 |
| S-c9 | 5.33E-05 | -0.07698 | 0.047505 | -0.10479 | 0.031202 | -0.01084 |
| S-c3ar | 0.000993 | 0.019554 | 0.0011 | 0.047811 | -0.0355 | -0.0145 |
| S-c5ar | 0.011905 | 0.018495 | -0.02499 | 0.005604 | 0.014165 | 0.000624 |
| S-cfi | -0.01299 | -0.07317 | -0.00171 | -0.00496 | 0.002105 | -0.00382 |
| S-cfh | -0.00346 | -0.02587 | 0.008825 | -0.08948 | -0.00174 | 0.005577 |
| S-serpin1 | -0.1445 | -0.08352 | 0.10609 | 0.033609 | 0.029842 | -0.03636 |
| S-cd59-1 | 0.002235 | ###### | 0.008673 | -0.02 | -0.00093 | 0.004811 |
| S-cd59-2 | -0.03341 | -0.31599 | -0.11844 | -0.17701 | -0.02484 | 0.022521 |
| S-c4bp | -0.00646 | 0.018939 | -0.00036 | -0.02272 | -0.0115 | 0.004174 |
|  |  |  |  |  |  |  |

**Table S2** Details of loading plot between AT and CT.

|  | PC1 | PC2 | PC3 | PC4 | PC5 | PC6 |
| --- | --- | --- | --- | --- | --- | --- |
| B-crf | 0.071442 | -0.07282 | 0.064918 | 0.042939 | -0.02968 | 0.001974 |
| B-crhr | 0.086517 | 0.033435 | -0.05401 | 0.08648 | -0.03228 | 0.24507 |
| B-pomcα | 0.065409 | 0.004753 | -0.04694 | 0.14871 | -0.01934 | 0.35042 |
| B-pomcβ | 0.080528 | -0.01667 | 0.067003 | 0.15863 | 0.004211 | 0.10933 |
| B-mc2r | 0.11143 | 0.099784 | -0.00484 | -0.04581 | 0.020499 | 0.6702 |
| B-mrap2 | 0.059061 | -0.05816 | -0.0486 | -0.07329 | 0.095844 | 0.032919 |
| B-grα | 0.068732 | 0.031785 | -0.07893 | 0.057966 | 0.057282 | 0.011434 |
| B-grβ | 0.11039 | 0.053627 | -0.02387 | 0.020135 | -0.02126 | -0.04147 |
| B-mrα | 0.08925 | 0.039743 | -0.04547 | -0.04233 | 0.017383 | -0.01896 |
| B-mrβ | 0.10701 | 0.032314 | -0.01083 | 0.018004 | 0.047685 | 0.003718 |
| B-hsd11β1 | 0.047579 | 0.029369 | 0.062667 | 0.0505 | 0.13007 | -0.00554 |
| B-hsd11β2 | -0.0229 | 0.07903 | -0.093 | 0.020379 | -0.0597 | -0.05081 |
| B-star | 0.018714 | 0.071367 | 0.010893 | -0.13062 | -0.13592 | 0.007913 |
| B-hsd3β1 | 0.037477 | 0.088178 | -0.12338 | 0.019593 | -0.07647 | -0.06049 |
| B-cyp11a1 p450scc | 0.029535 | 0.039485 | 0.10794 | 0.10491 | 0.035704 | 0.001582 |
| B-5ht1aα | 0.045231 | -0.02066 | 0.040738 | 0.079295 | -0.11558 | -0.0572 |
| B-5ht1aβ | 0.11096 | 0.019017 | -0.02627 | -0.00812 | -0.01332 | -0.00135 |
| B-sertα | -0.00642 | -0.07945 | -0.09057 | 0.074602 | 0.10603 | -0.06746 |
| B-sertβ | 0.093074 | -0.03461 | -0.07562 | 0.010254 | -0.02858 | -0.00494 |
| B-tph1a | 0.045455 | 0.018258 | -0.04489 | 0.06255 | -0.1247 | 0.043458 |
| B-tph2 | 0.086228 | 0.040537 | -0.00118 | -0.03074 | -0.06503 | -0.06489 |
| B-th | 0.047984 | 0.071424 | -0.04433 | 0.045598 | 0.088945 | -0.02804 |
| B-il1β1 | 0.013945 | 0.117 | 0.077992 | 0.058531 | 0.055748 | -0.01484 |
| B-il1β2 | -0.04282 | 0.053654 | -0.0171 | 0.036428 | 0.15489 | 0.086694 |
| B-il1β3 | 0.098264 | 0.07655 | -0.02289 | 0.002891 | -0.03025 | 0.070041 |
| B-il2 | 0.053215 | 0.10771 | 0.041868 | 0.074609 | 0.008892 | 0.01727 |
| B-il4 | 0.074359 | 0.001355 | -0.01461 | -0.02276 | 0.14075 | -0.0318 |
| B-il6 | 0.073564 | 0.015893 | -0.03447 | -0.05674 | 0.085174 | -0.03461 |
| B-il6b | 0.042458 | -0.04012 | -0.07153 | -0.0438 | 0.12604 | 0.007527 |
| B-il8 | -0.02878 | 0.1143 | 0.13415 | 0.004671 | 0.038156 | -0.02006 |
| B-il10b | -0.01756 | -0.07509 | -0.00855 | -0.02474 | 0.16107 | -0.01707 |
| B-il11 | 0.042122 | 0.06439 | 0.0277 | -0.13194 | 0.060432 | -0.02504 |
| B-tnfα1 | 0.053215 | 0.10771 | 0.041868 | 0.074609 | 0.008892 | -0.01071 |
| B-tnfα2 | 0.11774 | -0.05997 | -0.00409 | -0.01205 | 0.013353 | -0.04597 |
| B-tnfα3 | 0.074733 | 0.078026 | -0.09912 | -0.04678 | -0.03946 | -0.05091 |
| B-sod1 | 0.043502 | 0.067558 | 0.004308 | 0.052993 | -0.10335 | 0.024837 |
| B-sod2 | 0.061906 | 0.091854 | -0.08974 | 0.080795 | -0.07271 | 0.04589 |
| B-sod3 | -0.0605 | 0.043193 | -0.08156 | 0.096889 | 0.090676 | -0.0699 |
| B-cat | 0.10192 | 0.057406 | -0.01704 | 0.028351 | -0.00227 | -0.00507 |
| B-gsr | 0.072167 | 0.075274 | -0.10566 | 0.050748 | -0.04021 | -0.04964 |
| B-clec14a | 0.091084 | 0.039575 | -0.07343 | 0.03553 | ###### | 0.021302 |
| B-cd93 | -0.05679 | -0.03938 | -0.09827 | 0.093197 | -0.01948 | 0.031769 |
| B-clec4m | 0.057844 | 0.034685 | -0.04758 | 0.1212 | -0.00397 | -0.0025 |
| B-c1q | 0.078761 | 0.069908 | 0.058152 | -0.00753 | -0.03176 | 0.016216 |
| B-c1r/c1s | -0.07853 | 0.10473 | -0.00791 | 0.039623 | 0.051406 | 0.035926 |
| B-c2-b | -0.01655 | -0.10507 | -0.00761 | 0.08282 | -0.13027 | -0.01288 |
| B-cfb2 | -0.03748 | 0.011659 | -0.02551 | 0.000549 | 0.16261 | -0.00301 |
| B-c4 | -0.03036 | -0.05236 | -0.13386 | -0.02114 | 0.021406 | 0.046125 |
| B-c3-1b1 | 0.082908 | 0.006843 | 0.036518 | -0.04528 | -0.07034 | 0.043999 |
| B-c3-1b2 | 0.036348 | 0.12243 | 0.006641 | 0.097512 | -0.02504 | -0.10868 |
| B-c3-1a | 0.044169 | -0.10928 | 0.01421 | 0.091606 | -0.00402 | 0.031495 |
| B-c3-3 | -0.06636 | 0.015037 | 0.027676 | -0.0509 | -0.15205 | 0.051201 |
| B-c3-4 | -0.02322 | 0.006136 | 0.0807 | -0.01335 | 0.12408 | 0.015958 |
| B-masp2 | -0.03748 | 0.011659 | -0.02551 | 0.000549 | 0.16261 | -0.00301 |
| B-masp3 | 0.036545 | -0.03511 | 0.084224 | -0.03727 | 0.12499 | -0.04745 |
| B-mbl-h2 | 0.075791 | -0.00892 | 0.11483 | -0.04846 | 0.009281 | -0.00643 |
| B-cfp1 | -0.03694 | 0.006441 | 0.096785 | -0.0795 | -0.10014 | -0.00539 |
| B-cfp2 | -0.0207 | -0.00447 | 0.044033 | 0.14543 | 0.10598 | -0.0095 |
| B-c5 | 0.002462 | -0.03619 | -0.03296 | 0.16598 | 0.062926 | -0.04337 |
| B-c6 | 0.083351 | 0.051379 | -0.03542 | 0.00883 | 0.08591 | 0.003743 |
| B-c7-1 | 0.062588 | -0.01678 | -0.07962 | 0.042053 | 0.089261 | 0.03595 |
| B-c7-2 | -0.05698 | 0.009512 | -0.06279 | 0.079283 | 0.11177 | 0.011848 |
| B-c8 | -0.02392 | 0.029966 | -0.08783 | 0.13917 | 0.030415 | -0.02669 |
| B-c8b | 0.063463 | 0.03019 | -0.0613 | -0.04578 | 0.074095 | -0.03633 |
| B-c8g | 0.005726 | -0.10291 | -0.0208 | -0.10595 | -0.05732 | 0.034851 |
| B-c9 | 0.01762 | -0.04808 | -0.10492 | 0.085494 | 0.08039 | 0.075882 |
| B-c3ar | 0.046219 | 0.072573 | -0.08342 | 0.010452 | 0.12433 | -0.03202 |
| B-c5ar | 0.019183 | 0.12443 | -0.00689 | -0.02307 | 0.029574 | -0.00041 |
| B-cfi | 0.0095 | -0.12321 | 0.0837 | 0.045536 | -0.06219 | 0.042622 |
| B-cfh | 0.002337 | 0.12754 | -0.02459 | 0.088277 | 0.064941 | -0.05214 |
| B-serpin1 | -0.04617 | -0.0421 | -0.11337 | -0.07792 | 0.013508 | 0.040207 |
| B-cd59-1 | 0.052917 | 0.029496 | -0.09689 | 0.071814 | -0.08886 | -0.02067 |
| B-cd59-2 | -0.01576 | 0.056762 | -0.01416 | 0.046916 | -0.15571 | 0.008545 |
| B-c4bp | 0.023327 | 0.015383 | -0.09018 | 0.00886 | -0.11667 | -0.02633 |
| K-crhr | -0.01166 | -0.04215 | 0.037459 | 0.137 | -0.00995 | -0.02448 |
| K-pomcα | 0.066266 | -0.05039 | 0.055881 | 0.13988 | 0.001578 | 0.029501 |
| K-pomcβ | 0.053215 | 0.10771 | 0.041868 | 0.074609 | 0.008892 | 0.016982 |
| K-mc2r | -0.03694 | 0.006441 | 0.096785 | -0.0795 | -0.10014 | 0.057562 |
| K-mrap2 | 0.075352 | 0.11247 | -0.0287 | -0.06006 | 0.003708 | -0.09188 |
| K-grα | 0.002554 | 0.11912 | -0.10689 | 0.021727 | 0.016225 | -0.02264 |
| K-grβ | -0.02092 | -0.09067 | -0.07969 | 0.06342 | -0.10738 | -0.03676 |
| K-mrα | -0.0186 | 0.129 | 0.018222 | 0.038829 | 0.01471 | 0.00712 |
| K-mrβ | 0.036038 | 0.081457 | 0.077669 | 0.093976 | 0.041817 | 0.014763 |
| K-hsd11β1 | 0.053506 | 0.050938 | 0.04843 | 0.0013 | 0.13634 | -0.05796 |
| K-hsd11β2 | 0.078007 | 0.068364 | 0.041828 | 0.00209 | -0.03955 | -0.04927 |
| K-star | 0.10599 | -0.01506 | 0.043091 | -0.04082 | -0.01737 | 0.014625 |
| K-hsd3β1 | 0.082512 | 0.008853 | 0.016564 | -0.07265 | 0.042304 | -0.05095 |
| K-cyp21 | 0.072412 | 0.00173 | -0.05679 | -0.11349 | 0.011911 | -0.00589 |
| K-cyp11a1 p450scc | 0.072412 | 0.00173 | -0.05679 | -0.11349 | 0.011911 | -0.00589 |
| K-5ht1aα | 0.0207 | 0.12647 | -0.00444 | -0.01159 | -0.02268 | 0.020173 |
| K-5ht1aβ | -0.01771 | 0.031636 | -0.12546 | 0.052567 | -0.08471 | -0.01841 |
| K-sertα | 0.063087 | 0.082508 | 0.013888 | 0.090574 | -0.01992 | -0.02815 |
| K-sertβ | 0.1112 | 0.099205 | -0.0051 | -0.04632 | 0.020463 | 0.00977 |
| K-tph1a | 0.045607 | -0.08565 | -0.04009 | 0.037114 | -0.09315 | -0.01524 |
| K-th | 0.093397 | 0.056769 | 0.030477 | 0.009797 | -0.02386 | -0.03139 |
| K-il1β1 | 0.075034 | -0.09385 | 0.03855 | 0.031503 | 0.00127 | 0.014142 |
| K-il1β2 | 0.069378 | -0.00985 | 0.090953 | 0.11662 | 0.040268 | -0.0294 |
| K-il1β3 | 0.10364 | -0.06943 | -0.01466 | -0.02501 | -0.01244 | -0.03204 |
| K-il2 | 0.076492 | -0.06277 | -0.03024 | 0.068602 | -0.04805 | 0.043529 |
| K-il4 | 0.093561 | 0.044127 | 0.012874 | -0.02085 | -0.06099 | -0.06546 |
| K-il6 | 0.028685 | -0.04242 | 0.001758 | 0.11127 | 0.12769 | 0.034782 |
| K-il6b | 0.060294 | -0.12809 | -0.00798 | 0.014609 | 0.045615 | -0.01861 |
| K-il8 | 0.075333 | -0.03202 | 0.087241 | 0.089882 | -0.03385 | 0.009845 |
| K-il10b | 0.091408 | -0.02601 | 0.02458 | 0.104 | 0.018982 | -0.01468 |
| K-il11 | 0.093866 | -0.06364 | 0.043811 | 0.03098 | 0.023428 | -0.05963 |
| K-tnfα1 | 0.088442 | -0.13389 | -0.0115 | -0.03784 | 0.017005 | 0.040656 |
| K-tnfα2 | 0.055587 | -0.12622 | 0.018338 | 0.046003 | 0.00293 | -0.00886 |
| K-tnfα3 | 0.087547 | -0.089 | 0.005584 | 0.015796 | -0.02178 | -0.06066 |
| K-sod1 | -0.04546 | 0.085171 | -0.06973 | 0.008923 | -0.03981 | 0.025209 |
| K-sod2 | -0.05733 | 0.13089 | 0.025984 | 0.007404 | -0.07422 | -0.04835 |
| K-sod3 | 0.017118 | 0.10416 | 0.048048 | 0.04104 | -0.0962 | -0.02708 |
| K-cat | -0.01386 | 0.072426 | -0.12419 | 0.042022 | 0.001627 | -0.0058 |
| K-gsr | 0.061362 | -0.06252 | -0.03266 | -0.07347 | 0.11644 | 0.016304 |
| K-clec14a | 0.0171 | 0.075042 | 0.012664 | 0.13572 | -0.09451 | 0.002081 |
| K-cd93 | -0.10244 | 0.003107 | 0.016201 | 0.056512 | -0.00769 | 0.079999 |
| K-clec4m | -0.04036 | -0.12276 | 0.021673 | -0.07311 | 0.054485 | -0.03236 |
| K-c1q | 0.085645 | 0.029188 | 0.037046 | -0.04753 | 0.070539 | -0.01737 |
| K-c1r/c1s | -0.00386 | 0.1152 | 0.022544 | -0.08943 | 0.087421 | 0.016683 |
| K-c2-b | 0.012541 | 0.020178 | -0.16914 | -0.08294 | 0.016827 | 0.011384 |
| K-cfb2 | 0.047398 | 0.096257 | -0.12283 | 0.014734 | -0.07383 | -0.01294 |
| K-c4 | 0.1031 | -0.08774 | -0.00454 | -0.0421 | 0.013279 | -0.04247 |
| K-c3-1b1 | 0.10923 | -0.03127 | 0.01078 | -0.03985 | 0.002556 | -0.00319 |
| K-c3-1b2 | 0.08014 | 0.014204 | 0.057222 | 0.00258 | 0.097434 | -0.01403 |
| K-c3-1a | 0.11839 | -0.01246 | -0.0246 | 0.040077 | -0.03621 | -0.07675 |
| K-c3-3 | 0.13827 | -0.0125 | 0.019569 | 0.034004 | 0.016418 | 0.045074 |
| K-c3-4 | 0.05328 | -0.02407 | 0.093763 | -0.05529 | -0.10377 | -0.04117 |
| K-masp2 | -0.0348 | 0.034054 | -0.08325 | 0.15234 | 0.078249 | -0.05975 |
| K-masp3 | -0.04008 | 0.051144 | -0.07302 | 0.068625 | 0.11178 | 0.034383 |
| K-mbl-h2 | 0.075447 | 0.003613 | 0.096948 | 0.001501 | 0.026858 | -0.01401 |
| K-cfp1 | 0.027826 | 0.015612 | 0.096189 | -0.13704 | -0.03916 | 0.035606 |
| K-cfp2 | 0.050349 | 0.11561 | 0.055496 | -0.00364 | -0.01274 | -0.00972 |
| K-c5 | 0.009477 | -0.02103 | 0.16514 | 0.046898 | 0.039968 | 0.040084 |
| K-c6 | -0.04293 | -0.07499 | -0.04386 | -0.00444 | -0.14106 | 0.051065 |
| K-c7-1 | 0.069938 | 0.080188 | 0.066545 | -0.02706 | 0.022802 | -0.03438 |
| K-c7-2 | -0.11256 | 0.002628 | -0.03916 | -0.00673 | -0.0754 | -0.01623 |
| K-c8 | 0.031342 | 0.012072 | -0.13217 | -0.0795 | 0.025994 | -0.02619 |
| K-c8b | 0.044169 | -0.10928 | 0.01421 | 0.091606 | -0.00402 | 0.031782 |
| K-c8g | 0.046771 | 0.11317 | 0.066738 | 0.012448 | 0.024594 | -0.02302 |
| K-c9 | 0.099873 | 0.034491 | -0.04948 | 0.018248 | -0.05796 | 0.010685 |
| K-c3ar | 0.063929 | -0.13719 | 0.003848 | -0.00925 | 0.012602 | 0.016797 |
| K-c5ar | 0.02752 | -0.09448 | 0.006807 | -0.04066 | 0.15917 | 0.055253 |
| K-cfi | 0.030013 | 0.089712 | 0.1131 | -0.00659 | -0.07302 | -0.08173 |
| K-cfh | 0.007675 | 0.085083 | 0.090703 | 0.068787 | -0.09275 | -0.01861 |
| K-serpin1 | 0.053731 | -0.08958 | -0.0418 | 0.035422 | 0.06394 | -0.0231 |
| K-cd59-1 | -0.00775 | 0.10387 | -0.07876 | -0.01947 | 0.10453 | 0.078944 |
| K-cd59-2 | 0.003152 | 0.1263 | -0.05207 | -0.06715 | 0.075357 | 0.017591 |
| K-c4bp | 0.023734 | 0.13089 | -0.04337 | 0.034389 | 0.01563 | -0.01235 |
| S-crf | 0.076157 | -0.08147 | -0.02342 | 0.063698 | -0.02361 | -0.04762 |
| S-crhr | 0.076771 | 0.036871 | -0.11046 | 0.050966 | -0.04143 | 0.051507 |
| S-mc2r | -0.03694 | 0.006441 | 0.096785 | -0.0795 | -0.10014 | 0.078618 |
| S-mrap1 | -0.03694 | 0.006441 | 0.096785 | -0.0795 | -0.10014 | 0.078618 |
| S-mrap2 | 0.009959 | -0.08256 | -0.10024 | 0.13468 | -0.08884 | -0.03455 |
| S-grα | 0.056492 | 0.09498 | -0.09234 | 0.053081 | -0.01718 | -0.01559 |
| S-grβ | -0.09105 | 0.061303 | -0.0308 | -0.06496 | 0.00939 | 0.092189 |
| S-mrα | -0.09729 | 0.046907 | -0.0642 | -0.0295 | -0.01193 | 0.003718 |
| S-mrβ | -0.1063 | 0.045461 | -0.04864 | -0.04112 | -0.02522 | -0.03185 |
| S-hsd11β1 | 0.047128 | -0.06084 | 0.050671 | 0.042358 | 0.11808 | -0.02461 |
| S-hsd11β2 | -0.08587 | -0.00421 | -0.08573 | 0.036275 | -0.0348 | -0.05699 |
| S-star | -0.04721 | 0.029451 | -0.05487 | 0.091988 | -0.0587 | 0.037145 |
| S-hsd3β1 | 0.058667 | 0.031094 | 0.090799 | 0.048676 | -0.06062 | -0.01969 |
| S-cyp21 | 0.051888 | 0.10627 | 0.097382 | -0.05898 | -0.05179 | 0.038727 |
| S-cyp11a1 p450scc | 0.098936 | -0.0262 | 0.094386 | 0.034675 | -0.03497 | 0.049965 |
| S-5ht1aα | -0.07502 | 0.067454 | -0.0524 | -0.04061 | 0.035612 | -0.00531 |
| S-sertα | 0.1142 | -0.019 | 0.015588 | 0.047466 | -0.03668 | -0.0249 |
| S-sertβ | 0.072412 | 0.00173 | -0.05679 | -0.11349 | 0.011911 | -0.03533 |
| S-tph1a | 0.001435 | 0.11823 | -0.0356 | -0.05837 | -0.11025 | 0.013312 |
| S-th | 0.087968 | 0.098561 | 0.017718 | -0.00395 | 0.040013 | -0.00856 |
| S-il1β1 | 0.09193 | 0.020701 | 0.029226 | -0.07059 | 0.011864 | 0.047008 |
| S-il1β2 | 0.09718 | 0.03484 | 0.011398 | -0.06789 | 0.024103 | -0.1036 |
| S-il1β3 | 0.12124 | -0.01256 | 0.019937 | 0.015754 | -0.02271 | -0.02551 |
| S-il2 | 0.061917 | 0.036319 | -0.02739 | 0.07301 | 0.073052 | -0.01966 |
| S-il4 | 0.08806 | 0.024656 | 0.023292 | 0.099451 | 1.72E-05 | -0.03873 |
| S-il6 | 0.098217 | 0.044555 | -0.02148 | -0.03939 | 0.035463 | -0.03752 |
| S-il6b | 0.074049 | -0.01523 | -0.03597 | -0.11756 | 0.020652 | -0.01958 |
| S-il8 | 0.094322 | 0.00607 | -0.0014 | -0.07766 | 0.00063 | -0.06219 |
| S-il10b | 0.066156 | -0.03127 | -0.09104 | -0.08798 | -0.02082 | -0.01958 |
| S-il11 | 0.070792 | -0.00945 | -0.047 | -0.12205 | -0.00037 | -0.13245 |
| S-tnfα1 | 0.089751 | 0.003788 | -0.02557 | -0.08939 | 0.008541 | -0.03337 |
| S-tnfα2 | 0.087038 | 0.003787 | -0.02269 | -0.09715 | 0.007803 | 0.023013 |
| S-tnfα3 | 0.11167 | -0.00271 | 0.017729 | 0.013628 | -0.04468 | -0.03286 |
| S-sod1 | -0.01968 | 0.13671 | -0.05645 | 0.002691 | 0.013005 | -0.01515 |
| S-sod2 | 0.094578 | 0.059597 | 0.020959 | 0.029444 | -0.01291 | -0.01968 |
| S-sod3 | 0.002191 | 0.037086 | -0.11195 | 0.07395 | -0.04078 | 0.04679 |
| S-cat | -0.07712 | -0.01311 | -0.04619 | 0.00725 | 0.10381 | -0.00177 |
| S-gsr | 0.090135 | 0.04939 | 0.006749 | -0.08177 | -0.01277 | 0.042948 |
| S-clec14a | 0.081252 | -0.07091 | -0.01811 | 0.055533 | -0.03544 | -0.04978 |
| S-cd93 | 0.043383 | -0.00668 | -0.1597 | -0.03127 | -0.06324 | -0.03885 |
| S-clec4m | 0.075041 | 0.006028 | 0.079462 | -0.06001 | 0.029214 | 0.012644 |
| S-c1q | 0.059299 | 0.024496 | 0.10707 | 0.013611 | 0.011146 | -0.05376 |
| S-c1r/c1s | 0.035937 | -0.03854 | -0.14616 | 0.011952 | -0.01054 | -0.00303 |
| S-c2-b | 0.083964 | -0.07799 | -0.06445 | -0.0155 | 0.009291 | 0.040693 |
| S-cfb2 | 0.10032 | -0.03712 | -0.04688 | 0.008134 | 0.001809 | 0.042203 |
| S-c4 | 0.091121 | 0.054819 | -0.03572 | -0.07331 | 0.013025 | -0.04677 |
| S-c3-1b1 | 0.069641 | -0.02886 | -0.10095 | -0.05931 | -0.03626 | -0.0511 |
| S-c3-1b2 | 0.087729 | -0.06498 | -0.07489 | -0.02378 | -0.02893 | 0.039239 |
| S-c3-1a | 0.08743 | 0.024198 | -0.00998 | -0.06015 | -0.03093 | -0.03532 |
| S-c3-3 | 0.083264 | -0.0815 | -0.08348 | -0.02787 | -0.03327 | 0.10205 |
| S-c3-4 | 0.071506 | -0.0629 | -0.10184 | 0.008421 | -0.00708 | -0.01835 |
| S-masp2 | 0.017828 | 0.067352 | 0.053542 | 0.10722 | 0.13699 | 0.004508 |
| S-masp3 | 0.065724 | 0.04194 | -0.0149 | 0.082756 | 0.042481 | -0.04882 |
| S-mbl-h2 | 0.082632 | -0.08855 | 0.031478 | 0.030976 | 0.013967 | 0.025676 |
| S-cfp1 | 0.10062 | 0.028556 | -0.0213 | -0.03754 | -0.01503 | -0.02023 |
| S-cfp2 | -0.02411 | 0.036425 | -0.13252 | -0.0835 | -0.02412 | 0.062217 |
| S-c5 | -0.00092 | -0.02425 | -0.15422 | -0.06722 | -0.02417 | -0.00044 |
| S-c6 | -0.10701 | 0.007492 | 0.022051 | -0.05834 | 0.066501 | 0.048086 |
| S-c7-1 | 0.037433 | 0.035028 | -0.03202 | -0.10679 | 0.16948 | 0.064026 |
| S-c7-2 | -0.11522 | 0.036844 | -0.04174 | 0.000971 | 0.028041 | -0.02882 |
| S-c8 | 0.063303 | -0.09827 | -0.10567 | -0.02754 | -0.00776 | 0.024387 |
| S-c8b | 0.028935 | -0.07049 | -0.13187 | 0.054939 | -0.08726 | -0.08894 |
| S-c8g | -0.08496 | 0.072919 | -0.09779 | 0.061729 | 0.060113 | 0.00134 |
| S-c9 | 0.09306 | -0.04609 | -0.04636 | 0.054716 | -0.00578 | -0.00582 |
| S-c3ar | 0.053145 | 0.030419 | -0.01632 | -0.13821 | -0.01801 | 0.090911 |
| S-c5ar | 0.034478 | 0.011837 | 0.063939 | -0.11833 | 0.057889 | -0.0127 |
| S-cfi | 0.071842 | -0.12399 | -0.00499 | 0.024731 | 0.002311 | 0.044399 |
| S-cfh | 0.026681 | -0.06083 | -0.07769 | -0.09525 | -0.02188 | 0.047224 |
| S-serpin1 | -0.02328 | 0.000536 | -0.16547 | -0.02158 | 0.021517 | 0.005921 |
| S-cd59-1 | 0.10669 | 0.081152 | -0.03794 | -0.01919 | -0.01537 | -0.0036 |
| S-cd59-2 | 0.07358 | -0.02978 | 0.054989 | 0.064937 | -0.06487 | 0.0304 |
| S-c4bp | -0.00553 | 0.1304 | -0.00277 | -0.0349 | -0.06688 | -0.00392 |

**Table S3** Details of loading plot between AT and ST.

|  | PC1 | PC2 | PC3 | PC4 | PC5 | PC6 |
| --- | --- | --- | --- | --- | --- | --- |
| B-crf | 0.046644 | -0.04597 | 0.040468 | -0.02729 | -0.10628 | 0.18617 |
| B-crhr | 0.060761 | 0.024876 | 0.015251 | 0.020108 | -0.00406 | -0.33461 |
| B-pomcα | 0.091854 | -0.03578 | -0.04862 | 0.030734 | -0.08496 | 0.28347 |
| B-pomcβ | 0.051 | -0.10064 | -0.14152 | 0.098862 | -0.21095 | -0.68808 |
| B-mc2r | -0.00912 | -0.03133 | 0.034377 | 0.046183 | 0.052804 | 0.13415 |
| B-mrap1 | 0.029759 | 0.0262 | 0.006215 | -0.00507 | 0.012086 | 0.002045 |
| B-mrap2 | 0.017391 | -0.03931 | 0.050272 | -0.07912 | 0.042912 | 0.001692 |
| B-grα | 0.050561 | -0.0805 | 0.009319 | 0.011507 | 0.024238 | -0.03156 |
| B-grβ | 0.024011 | -0.05507 | 0.035523 | 0.041485 | 0.032882 | -0.03706 |
| B-mrα | -0.01333 | -0.11136 | -0.01681 | 0.016851 | 0.092511 | -0.06684 |
| B-mrβ | 0.013212 | -0.09931 | -0.04457 | 0.01907 | 0.089151 | 0.018951 |
| B-hsd11β1 | 0.004292 | -0.00732 | -0.02536 | 0.09816 | -0.03144 | 0.011206 |
| B-hsd11β2 | 0.002567 | -0.07178 | 0.032081 | 0.042305 | 0.000269 | -0.04531 |
| B-star | -0.0159 | -0.03176 | 0.065304 | 0.027007 | 0.051612 | -0.02168 |
| B-hsd3β1 | 0.005136 | 0.038771 | 0.055188 | 0.0347 | 0.028241 | -0.01809 |
| B-cyp21 | 0.033403 | 0.029409 | 0.006976 | -0.00569 | 0.013566 | -0.01619 |
| B-cyp11a1 p450scc | 0.048598 | 0.01595 | -0.05146 | 0.055544 | -0.00174 | 0.021478 |
| B-5ht1aα | 0.094626 | 0.033176 | -0.01813 | -0.01146 | -0.06011 | 0.016361 |
| B-5ht1aβ | 0.04687 | -0.03723 | 0.055544 | -0.00291 | -0.00895 | -0.00397 |
| B-sertα | 0.0658 | 0.000553 | -0.04027 | -0.06794 | 0.012868 | -0.01247 |
| B-sertβ | 0.038205 | -0.05651 | 0.067587 | -0.05515 | -0.02649 | -0.03134 |
| B-tph1a | 0.029795 | -0.01931 | 0.054782 | 0.019065 | -0.09366 | 0.014191 |
| B-tph2 | 0.078455 | -0.02357 | 0.064651 | 0.007909 | 0.034082 | 0.024827 |
| B-th | 0.075398 | -0.00992 | 0.06651 | 0.081726 | 0.013227 | 0.011595 |
| B-il1β1 | 0.014382 | 0.023652 | 0.016161 | 0.14112 | -0.03677 | -0.00421 |
| B-il1β2 | 0.013931 | 0.033165 | -0.02362 | 0.088715 | 0.029395 | 0.034486 |
| B-il1β3 | 0.015639 | -0.04312 | 0.069928 | 0.049444 | -0.00382 | 0.064894 |
| B-il2 | 0.025455 | 0.055233 | 0.030523 | 0.042135 | -0.0311 | 0.016613 |
| B-il4 | -0.01043 | -0.01211 | -0.01783 | -0.00551 | 0.11695 | -0.01474 |
| B-il6 | 0.025872 | -0.05519 | 0.051917 | -0.01113 | 0.081925 | -0.06101 |
| B-il6b | 0.0343 | -0.01961 | 0.027204 | -0.06734 | 0.065707 | -0.05053 |
| B-il8 | -0.02451 | -0.0022 | 0.052286 | 0.079021 | -0.08426 | -0.00419 |
| B-il10b | -0.0279 | 0.041669 | 0.003516 | -0.06422 | -0.02317 | 0.017774 |
| B-il11 | 0.001179 | 0.041559 | 0.029462 | 0.017052 | 0.12104 | -0.02474 |
| B-tnfα1 | -0.0159 | -0.014 | 0.007034 | 0.11173 | -0.03011 | 0.015766 |
| B-tnfα2 | -0.01759 | -0.05064 | 0.04732 | -0.03783 | -0.04286 | 0.036345 |
| B-tnfα3 | 0.005531 | 0.014975 | 0.10443 | 0.017898 | -0.00085 | 0.011111 |
| B-sod1 | 0.095146 | -0.04711 | 0.007835 | 0.028143 | 0.015014 | 0.019476 |
| B-sod2 | 0.091746 | -0.04578 | 0.018789 | 0.027755 | 0.0276 | -0.03765 |
| B-sod3 | 0.019444 | -0.06583 | -0.02366 | 0.029548 | -0.00937 | 0.014433 |
| B-cat | 0.035382 | -0.14301 | 0.032508 | 0.034121 | -0.0059 | -0.11867 |
| B-gsr | 0.062897 | -0.03591 | 0.085219 | 0.02847 | -0.03142 | 0.011655 |
| B-clec14a | 0.063946 | -0.04086 | 0.043368 | 0.010716 | 0.025563 | -0.02025 |
| B-cd93 | 0.057711 | -0.0159 | -0.00755 | -0.03436 | -0.04485 | 0.000516 |
| B-clec4m | -0.05558 | 0.016153 | 0.01808 | 0.046471 | -0.08551 | 0.004857 |
| B-c1q | 0.007865 | 0.022987 | 0.09556 | 0.028791 | -0.09771 | 0.024868 |
| B-c1r/c1s | -0.02009 | -0.01155 | -0.03459 | 0.071978 | 0.032949 | -0.01698 |
| B-c2-b | 0.005999 | 0.057176 | -0.02578 | -0.03861 | -0.0396 | -0.00409 |
| B-cfb2 | -0.00596 | -0.00953 | -0.04283 | 0.004886 | 0.063126 | 0.011209 |
| B-c4 | -0.03977 | 0.006354 | 0.056359 | -0.06566 | -0.04556 | 0.033018 |
| B-c3-1b1 | -0.07513 | 0.025369 | 0.021382 | 0.015997 | -0.00811 | -0.00081 |
| B-c3-1b2 | -0.01282 | -0.0222 | -0.05397 | 0.11973 | 0.049199 | -0.00619 |
| B-c3-1a | 0.016339 | 0.0463 | 0.010425 | -0.03345 | -0.07143 | 0.021313 |
| B-c3-3 | 0.030119 | 0.088436 | -0.01526 | -0.01289 | 0.04249 | 0.008582 |
| B-c3-4 | 0.029153 | -0.09868 | -0.03668 | 0.01391 | 0.036446 | 0.024655 |
| B-masp2 | 0.029759 | 0.0262 | 0.006215 | -0.00507 | 0.012086 | 0.002045 |
| B-masp3 | 0.049147 | 0.038393 | 0.026618 | -0.01986 | -0.01427 | 0.007807 |
| B-mbl-h2 | 0.03729 | 0.01352 | 0.00128 | 0.000314 | 0.040973 | -0.00354 |
| B-cfp1 | 0.029759 | 0.0262 | 0.006215 | -0.00507 | 0.012086 | 0.002045 |
| B-cfp2 | 0.049857 | 0.0121 | -0.0251 | 0.025839 | -0.09939 | 0.023473 |
| B-c5 | 0.020498 | -0.00838 | -0.09576 | 0.008001 | -0.0558 | 0.043226 |
| B-c6 | 0.045133 | -0.05914 | 0.098687 | 0.031486 | -0.03782 | 0.002982 |
| B-c7-1 | 0.046082 | -0.0559 | 0.043367 | -0.03875 | -0.01437 | 0.01878 |
| B-c7-2 | 0.07142 | -0.09294 | -0.00131 | 0.003333 | -0.0357 | 0.006971 |
| B-c8 | 0.027561 | 0.042566 | -0.02388 | 0.033268 | -0.0731 | 0.026125 |
| B-c8b | 0.040439 | 0.034943 | 0.023501 | -0.01338 | 0.088099 | -0.00452 |
| B-c8g | 0.001834 | 0.038476 | 0.042778 | -0.07452 | -0.01577 | -0.02154 |
| B-c9 | 0.051915 | -0.02709 | -0.03399 | -0.0428 | 0.017959 | -0.04337 |
| B-c3ar | -0.00233 | 0.010571 | 0.11039 | 0.03901 | -0.05331 | 0.001102 |
| B-c5ar | 0.010225 | 0.020018 | 0.056875 | 0.10123 | 0.062208 | 0.008044 |
| B-cfi | 0.028707 | 0.015402 | -0.06355 | -0.05358 | -0.01553 | 0.003537 |
| B-cfh | 0.025729 | 0.045511 | 0.002151 | 0.068908 | 0.011661 | -0.00526 |
| B-serpin1 | 0.050533 | 0.039782 | 0.030965 | -0.05363 | 0.02588 | -0.03628 |
| B-cd59-1 | 0.065118 | -0.07474 | -0.00858 | 0.001774 | 0.033167 | 0.014138 |
| B-cd59-2 | 0.093564 | -0.08367 | -0.00458 | 0.028013 | 0.008113 | -0.00215 |
| B-c4bp | 0.074709 | -0.05314 | 0.054322 | -0.02297 | 0.00239 | 0.045363 |
| K-crhr | 0.026072 | -0.03976 | -0.03649 | 0.003163 | -0.07439 | 0.014664 |
| K-pomcα | 0.026072 | -0.03976 | -0.03649 | 0.003163 | -0.07439 | 0.014664 |
| K-pomcβ | -0.01963 | -0.01383 | -0.02859 | 0.083737 | 0.024064 | -0.00478 |
| K-mc2r | -0.01555 | 0.000999 | 0.035826 | 0.00911 | -0.04342 | -0.00427 |
| K-mrap2 | 0.035558 | -0.00488 | 0.092679 | 0.064563 | 0.086555 | -0.02853 |
| K-grα | 0.050241 | -0.08904 | -0.00423 | 0.037569 | 0.07608 | 0.030118 |
| K-grβ | -0.01188 | -0.1027 | -0.0379 | -0.03426 | -0.02119 | 0.038064 |
| K-mrα | 0.033692 | -0.10092 | 0.044771 | 0.094242 | -0.04091 | 0.000426 |
| K-mrβ | 0.053432 | -0.08696 | 3.79E-05 | 0.057413 | -0.02989 | 0.025102 |
| K-hsd11β1 | 0.028019 | -0.0386 | 0.011624 | 0.071136 | 0.045944 | 0.004401 |
| K-hsd11β2 | 0.060273 | -0.06716 | 0.053038 | 0.019494 | -0.03474 | -0.00939 |
| K-star | -0.10629 | 0.009562 | 0.021769 | 0.015723 | -0.02592 | 0.035035 |
| K-hsd3β1 | -0.07382 | 0.035983 | 0.066896 | 0.007984 | 0.007105 | 0.000275 |
| K-cyp21 | -0.00706 | -0.01834 | 0.073516 | -0.02234 | 0.017525 | -0.04262 |
| K-cyp11a1 p450scc | -0.01494 | 0.028738 | 0.086917 | -0.01755 | -0.02617 | 0.00139 |
| K-5ht1aα | 0.071067 | 0.036664 | 0.047585 | 0.062898 | 0.049967 | 0.010236 |
| K-sertα | 0.080186 | -0.04569 | -0.00217 | 0.067218 | 0.003903 | 0.040683 |
| K-sertβ | 0.020759 | -0.03693 | 0.045818 | 0.035335 | 0.035652 | -0.02161 |
| K-tph1a | 0.015031 | 0.052172 | -0.00885 | -0.10868 | -0.06117 | -0.01926 |
| K-th | -0.025 | -0.01864 | 0.11339 | 0.1894 | 0.010881 | 0.015069 |
| K-il1β1 | 0.11084 | 0.11798 | -0.03184 | -0.12833 | -0.03172 | 0.030807 |
| K-il1β2 | 0.090347 | 0.13052 | -0.03312 | 0.003659 | -0.02557 | 0.091324 |
| K-il1β3 | 0.046072 | -0.09068 | 0.071208 | -0.13202 | -0.00975 | 0.014518 |
| K-il2 | -0.02048 | -0.06794 | -0.04722 | -0.02508 | -0.00321 | 0.004914 |
| K-il4 | 0.050567 | 0.04871 | 0.03088 | 0.013283 | 0.05639 | -0.0379 |
| K-il6 | 0.068682 | 0.013342 | -0.11645 | -0.02194 | 0.08506 | -0.00656 |
| K-il6b | 0.031199 | -0.0828 | -0.00806 | -0.13991 | -0.06947 | 0.01381 |
| K-il8 | 0.059439 | 0.13247 | 0.00251 | -0.00264 | -0.12168 | -0.06465 |
| K-il10b | 0.041812 | 0.021273 | 0.049658 | 0.000601 | -0.13465 | 0.041719 |
| K-il11 | 0.07 | 0.12135 | 0.029538 | -0.08146 | -0.09106 | -0.06307 |
| K-tnfα1 | 0.065564 | -0.03744 | 0.025015 | -0.16264 | -0.00594 | 0.002577 |
| K-tnfα2 | 0.086943 | -0.02473 | -0.06225 | -0.18154 | -0.10304 | 0.01598 |
| K-tnfα3 | 0.054452 | 0.024176 | -0.00796 | -0.10099 | -0.01432 | 0.014653 |
| K-sod1 | 0.040402 | -0.07546 | 0.025085 | 0.090427 | 0.070328 | -0.01582 |
| K-sod2 | 0.1196 | -0.02921 | 0.027329 | 0.090609 | 0.030254 | -0.01502 |
| K-sod3 | 0.041926 | -0.10856 | 0.004405 | 0.047251 | -0.00272 | -0.00825 |
| K-cat | 0.11303 | -0.06815 | 0.024638 | 0.008795 | 0.042171 | -0.0562 |
| K-gsr | -0.11362 | -0.05919 | -0.02878 | -0.07754 | 0.12943 | -0.04202 |
| K-clec14a | 0.077394 | -0.05919 | -0.02243 | 0.010737 | 0.024811 | 0.042021 |
| K-cd93 | 0.061543 | -0.06245 | -0.07674 | 0.01491 | -0.01884 | -0.01636 |
| K-clec4m | -0.08474 | -0.10249 | -0.08948 | -0.0703 | 0.060957 | 0.028942 |
| K-c1q | 0.077171 | -0.11822 | -0.05833 | 0.008437 | 0.17829 | 0.075688 |
| K-c1r/c1s | 0.000655 | 0.01877 | 0.099556 | 0.065502 | 0.00597 | 0.021908 |
| K-c2-b | -0.04747 | -0.00238 | 0.063338 | -0.01262 | 0.009458 | -0.02026 |
| K-cfb2 | -0.04972 | -0.00753 | 0.030981 | 0.04316 | 0.020258 | -0.04086 |
| K-c4 | -0.02653 | -0.10762 | 0.026507 | -0.14611 | 0.030325 | -0.00814 |
| K-c3-1b1 | 0.066178 | 0.12167 | 0.049246 | -0.08381 | 0.094364 | -0.05745 |
| K-c3-1b2 | 0.06926 | 0.10788 | 0.017071 | -0.00941 | 0.022741 | -0.00077 |
| K-c3-1a | 0.074294 | 0.082954 | -0.01879 | -0.02892 | 0.067529 | -0.00498 |
| K-c3-3 | -0.03377 | 0.039722 | 0.021223 | 0.006768 | -0.02784 | -0.00904 |
| K-c3-4 | -0.00794 | -0.05085 | -0.00831 | -0.02387 | 0.040081 | -0.03762 |
| K-masp2 | -0.00437 | -0.07382 | -0.09504 | 0.03382 | 0.048836 | -0.00402 |
| K-masp3 | 0.036975 | 0.054296 | -0.03306 | 0.030472 | 0.045096 | -0.03089 |
| K-mbl-h2 | 0.060112 | 0.044924 | 0.030739 | 0.057507 | -0.04546 | 0.02869 |
| K-cfp1 | 0.023796 | -0.07833 | 0.10994 | 0.00453 | -0.00699 | -0.02372 |
| K-cfp2 | 0.17169 | 0.076271 | 0.077425 | 0.11572 | 0.070325 | -0.00487 |
| K-c5 | 0.14699 | 0.075416 | -0.07227 | 0.037909 | -0.14158 | 0.099906 |
| K-c6 | 0.056114 | -0.18876 | -0.12187 | -0.07717 | 0.11345 | 0.032085 |
| K-c7-1 | 0.13801 | 0.26462 | 0.045701 | -0.00579 | 0.060989 | 0.067743 |
| K-c7-2 | 0.073174 | -0.15735 | -0.08271 | -0.02655 | 0.057955 | 0.004133 |
| K-c8 | 0.063423 | 0.10583 | 0.032599 | -0.04387 | 0.07521 | -0.00312 |
| K-c8b | -0.00868 | 0.040018 | -0.01906 | -0.04802 | -0.09127 | 0.020192 |
| K-c8g | 0.10613 | 0.083501 | 0.066023 | 0.18793 | 0.048967 | -0.00224 |
| K-c9 | 0.034218 | -0.01958 | 0.077491 | 0.022749 | 0.013564 | -0.01739 |
| K-c3ar | 0.01082 | -0.08471 | -0.04246 | -0.17717 | -0.02225 | -0.05985 |
| K-c5ar | -0.00997 | -0.13262 | -0.06336 | -0.09075 | 0.077446 | -0.01604 |
| K-cfi | 0.10985 | 0.048113 | 0.019205 | 0.079985 | 0.032748 | -0.01672 |
| K-cfh | 0.22326 | 0.20111 | -0.01614 | 0.10549 | 0.00175 | -0.12438 |
| K-serpin1 | -0.02685 | -0.05999 | 0.00669 | -0.09516 | -0.0869 | 0.054651 |
| K-cd59-1 | 0.095759 | 0.041938 | 0.01937 | 0.018602 | 0.10512 | -0.04018 |
| K-cd59-2 | 0.040347 | 0.01226 | 0.060406 | 0.056204 | 0.10104 | -0.02779 |
| K-c4bp | 0.06634 | -0.04836 | 0.059356 | 0.10479 | 0.027339 | 0.023633 |
| S-crf | 0.047407 | -0.00421 | -0.05875 | -0.0715 | 0.016016 | 0.029791 |
| S-crhr | 0.056496 | -0.04383 | 0.053784 | 0.009564 | 0.019646 | 0.007418 |
| S-pomcα | 0.033771 | 0.029732 | 0.007052 | -0.00575 | 0.013716 | -0.01061 |
| S-mc2r | -0.00682 | -0.0109 | -0.04901 | 0.005591 | 0.072236 | -0.00394 |
| S-mrap1 | 0.002823 | 0.023231 | -0.05499 | -0.00121 | 0.083213 | 0.004449 |
| S-mrap2 | 0.039967 | 0.050896 | -0.01527 | -0.05564 | -0.06798 | -0.01731 |
| S-grα | 0.058099 | -0.11598 | 0.033567 | 0.027111 | -0.00145 | 0.002861 |
| S-grβ | 0.02895 | -0.04853 | 0.029888 | 0.04896 | 0.12932 | -0.03895 |
| S-mrα | 0.073775 | 0.047975 | 0.017014 | -0.00587 | 0.053843 | 0.061132 |
| S-mrβ | 0.059745 | 0.062909 | 0.030699 | 0.001292 | 0.047443 | -0.02963 |
| S-hsd11β1 | 0.062025 | -0.0453 | -0.02233 | -0.04145 | -0.05775 | 0.019911 |
| S-hsd11β2 | -0.00104 | 0.032756 | -0.03224 | -0.03691 | -0.04544 | -0.02598 |
| S-star | -0.04847 | 0.021195 | -0.12029 | 0.062359 | 0.007764 | 0.0208 |
| S-hsd3β1 | -0.08757 | -0.00957 | -0.11926 | 0.07659 | 0.071946 | 0.01942 |
| S-cyp21 | 0.044632 | 0.000391 | 0.040621 | 0.056081 | 0.032634 | 0.001234 |
| S-cyp11a1 p450scc | 0.015846 | -0.02656 | 0.027595 | 0.009653 | -0.09112 | 0.022677 |
| S-5ht1aα | -0.11621 | 0.063646 | -0.07654 | 0.034657 | 0.10904 | -0.00975 |
| S-5ht1aβ | -0.0163 | 0.001047 | 0.037539 | 0.009546 | -0.0455 | -0.0033 |
| S-sertα | 0.05208 | -0.04445 | -0.00076 | -0.01004 | -0.02945 | -0.02672 |
| S-sertβ | 0.015071 | -0.03268 | 0.061164 | -0.03081 | 0.060191 | -0.03413 |
| S-tph1a | -0.06662 | 0.009677 | 0.037712 | 0.088592 | 0.082342 | -0.06208 |
| S-tph2 | 0.050737 | 0.044669 | 0.010595 | -0.00864 | 0.020606 | -0.00738 |
| S-th | 0.020822 | -0.05515 | 0.003533 | 0.12244 | 0.15024 | 0.087225 |
| S-il1β1 | -0.22389 | 0.071182 | 0.10537 | 0.054913 | -0.03698 | 0.037522 |
| S-il1β2 | -0.16058 | 0.074732 | 0.11916 | 0.042902 | -0.10589 | -0.03865 |
| S-il1β3 | 0.007159 | -0.09378 | 0.12833 | 0.005883 | -0.14577 | 0.026182 |
| S-il2 | 0.056011 | -0.02975 | 0.065876 | 0.041707 | -0.08104 | 0.004893 |
| S-il4 | 0.058639 | -0.01364 | 0.007898 | 0.041155 | -0.05749 | 0.031721 |
| S-il6 | -0.11945 | -0.0234 | 0.16072 | 0.063454 | -0.05739 | -0.07257 |
| S-il6b | -0.0746 | -0.03394 | 0.29297 | -0.11972 | 0.071104 | 0.017688 |
| S-il8 | -0.1839 | 0.049807 | 0.12378 | 0.015927 | -0.02156 | -0.07358 |
| S-il10b | -0.00644 | 0.002672 | 0.079266 | -0.1024 | 0.11676 | -0.04924 |
| S-il11 | -0.21962 | 0.029231 | 0.080261 | -0.02927 | 0.10059 | 0.08061 |
| S-tnfα1 | -0.11056 | -0.00457 | 0.20213 | -0.01577 | -0.0414 | -0.06251 |
| S-tnfα2 | -0.07982 | 0.001826 | 0.21032 | -0.03639 | 0.031309 | -0.00213 |
| S-tnfα3 | -0.06921 | -0.02318 | 0.090919 | 0.025677 | -0.11276 | 0.002124 |
| S-sod1 | -0.00053 | -0.01893 | -0.06006 | 0.091421 | 0.17734 | -0.0583 |
| S-sod2 | 0.079485 | -0.0296 | 0.007165 | 0.047283 | 0.056287 | -0.00227 |
| S-sod3 | 0.084972 | -0.051 | 0.065968 | 0.01015 | -0.06624 | 0.039298 |
| S-cat | 0.046467 | -0.09569 | -0.05827 | -0.02143 | 0.064138 | -0.02727 |
| S-gsr | -0.01278 | -0.01996 | 0.13564 | 0.041725 | 0.095803 | -0.00712 |
| S-clec14a | 0.002209 | -0.12891 | 0.009828 | -0.02433 | -0.06683 | -0.08439 |
| S-cd93 | 0.01706 | -0.11282 | 0.059974 | -0.01497 | -0.03399 | 0.061454 |
| S-clec4m | 0.084224 | -0.07071 | 0.14614 | -0.00127 | -0.11746 | -0.00479 |
| S-c1q | 0.10918 | -0.18843 | 0.087372 | 0.053233 | -0.1269 | 0.04706 |
| S-c1r/c1s | 0.033549 | -0.03323 | 0.066753 | -0.12296 | 0.018251 | -0.06842 |
| S-c2-b | 0.059867 | -0.02137 | 0.028006 | -0.11147 | 0.031487 | -0.00808 |
| S-cfb2 | 0.082877 | -0.02212 | 0.070494 | -0.07245 | -0.01111 | -0.03827 |
| S-c4 | 0.058227 | -0.05785 | 0.26302 | 0.024901 | -0.03315 | 0.011457 |
| S-c3-1b1 | -0.01627 | 0.091217 | 0.096498 | -0.12504 | 0.11744 | -0.10815 |
| S-c3-1b2 | 0.048138 | 0.042677 | 0.052454 | -0.12692 | 0.042138 | 0.003511 |
| S-c3-1a | -0.05628 | 0.055487 | 0.04851 | 0.009202 | 0.039397 | 0.046984 |
| S-c3-3 | 0.000353 | 0.005598 | 0.025929 | -0.18154 | 0.079539 | -0.05088 |
| S-c3-4 | 0.075997 | 0.020805 | 0.058188 | -0.10226 | -0.00768 | -0.01371 |
| S-masp2 | 0.01437 | -0.03429 | 0.000352 | 0.093075 | -0.07852 | 0.042844 |
| S-masp3 | 0.075385 | 0.029349 | 0.03572 | 0.033352 | -0.04113 | -0.01507 |
| S-mbl-h2 | -0.1474 | -0.13871 | -0.06476 | -0.12244 | -0.17009 | -0.03116 |
| S-cfp1 | 0.059272 | -0.03112 | 0.12054 | 0.004756 | 0.082352 | -0.02196 |
| S-cfp2 | 0.028949 | -0.06458 | 0.090818 | -0.03436 | 0.10969 | -0.04774 |
| S-c5 | 0.017887 | -0.01922 | 0.1516 | -0.14979 | 0.068707 | 0.001694 |
| S-c6 | -0.15726 | -0.10908 | -0.09685 | 0.040078 | 0.080325 | 0.057066 |
| S-c7-1 | -0.23295 | 0.11175 | -0.04321 | 0.040317 | 0.056954 | -0.10164 |
| S-c7-2 | 0.022076 | -0.17667 | 0.027094 | 0.074294 | -0.07471 | 0.035413 |
| S-c8 | 0.037903 | -0.00713 | 0.036566 | -0.14599 | 0.032479 | 0.005017 |
| S-c8b | 0.056477 | 0.047575 | 0.005977 | -0.06505 | -0.00252 | 0.014925 |
| S-c8g | -0.00214 | -0.0816 | -0.04258 | 0.076038 | 0.038454 | 0.04002 |
| S-c9 | 0.092789 | -0.01502 | -0.04044 | -0.08081 | 0.049917 | 0.010229 |
| S-c3ar | -0.01553 | -0.05721 | 0.17711 | -0.0093 | 0.026753 | -0.02417 |
| S-c5ar | -0.05532 | -0.05159 | 0.055821 | 0.011845 | 0.086379 | -0.0136 |
| S-cfi | 0.04138 | -0.00553 | -0.00499 | -0.08307 | -0.03252 | 0.013007 |
| S-cfh | 0.009327 | -0.00361 | 0.031808 | -0.17434 | 0.17589 | -0.01853 |
| S-serpin1 | 0.084536 | -0.00207 | 0.09693 | -0.09507 | 0.066739 | -0.08014 |
| S-cd59-1 | 0.020406 | -0.06766 | 0.046143 | 0.062775 | 0.11481 | -0.03163 |
| S-cd59-2 | 0.12005 | 0.17963 | -0.00696 | -0.04072 | 0.041741 | -0.03373 |
| S-c4bp | -0.02263 | -0.0609 | 0.00116 | 0.10863 | 0.10561 | 0.006855 |
